# Selective recognition and discrimination of single isomeric changes in peptide strands with a host : guest sensing array†

**DOI:** 10.1039/d3sc06087j

**Published:** 2024-01-02

**Authors:** Junyi Chen, Parisa Fasihianifard, Alexie Andrea P. Raz, Briana L. Hickey, Jose L. Moreno, Chia-En A. Chang, Richard J. Hooley, Wenwan Zhong

**Affiliations:** a Department of Chemistry, University of California–Riverside Riverside CA 92521 USA wenwan.zhong@ucr.edu richard.hooley@ucr.edu; b Environmental Toxicology Graduate Program, University of California–Riverside Riverside CA 92521 USA

## Abstract

An indirect competitive binding mechanism can be exploited to allow a combination of cationic fluorophores and water-soluble synthetic receptors to selectively recognize and discriminate peptide strands containing a single isomeric residue in the backbone. Peptide isomerization occurs in long-lived proteins and has been linked with diseases such as Alzheimer's, cataracts and cancer, so isomers are valuable yet underexplored targets for selective recognition. Planar cationic fluorophores can selectively bind hydrophobic, Trp-containing peptide strands in solution, and when paired with receptors that provide a competitive host for the fluorophore, can form a differential sensing array that enables selective discrimination of peptide isomers. Residue variations such as D- and L-Asp, D- and L-isoAsp, D-Ser and D-Glu can all be recognized, simply by their effects on the folded structure of the flexible peptide. Molecular dynamics simulations were applied to determine the most favorable conformation of the peptide : fluorophore conjugate, indicating that favorable π-stacking with internal tryptophan residues in a folded binding pocket enables micromolar binding affinity.

## Abstract 2

A host : indicator array comprising cationic fluorophores and water-soluble receptors can selectively discriminate peptides containing a single isomeric residue in the backbone.

## Graphical abstract

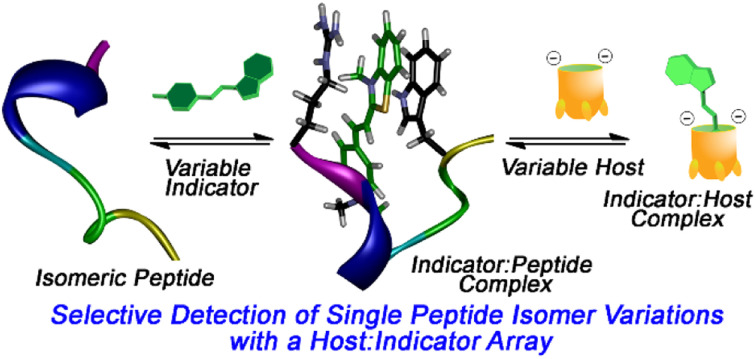

## Introduction

Post-translational modifications of peptides and proteins underpin the field of epigenetics, and can have wide-ranging downstream effects on protein structure, function, stability, molecular interaction and/or subcellular localization.^1^ Modifications include methylation, acetylation, and phosphorylation, among others.^2^ There is another type of modification that is less studied: peptide isomerization, *i.e.* peptides with one or more residues substituted by their rearranged isomers or d-amino acids.^3^ Epimerization, which occurs when a single amino acid undergoes stereoinversion, is an important modification that occurs as a function of aging, especially for long-lived proteins,^4^*e.g.*, amyloid-beta (Aβ), Tau, and crystallin proteins.^5^d-amino acid-containing peptides have been linked with diseases such as Alzheimer's, cataracts and cancer.^6^ Additionally, isomerization of aspartate residues (isoAsp) occurs along with epimerization in the crystallin of human eye lens, due to the absence of protein turnover.^7^ The accumulation of Asp isomerization perturbs protein structure, decreases crystallin solubility and lens transparency, ultimately leading to cataracts.^8^

Detecting these modifications remains a challenge, as the structural differences between single isomers in an oligopeptide or protein are extremely small. The current approaches for the analysis of protein isomerization rely on mass spectrometry (MS) technology.^5*b*,*c*,7*b*,9^ LC-MS/MS using radical directed dissociation (RDD) and collision-induced dissociation (CID) have been combined to improve the separation and identification of isomers. However, these MS-based strategies usually require expensive instruments, labeling on amino acids, or complex ionization processes. As far as we are aware, there are no examples of using optical sensing processes to detect and discriminate single isomers of oligopeptides. Amino acids can be chiroptically detected by binding in species such as modified cucurbiturils,^10^ but oligopeptides are a far more challenging target.

Differential sensing^11^ is a powerful tool for detecting small changes in structure for different biological targets, including oligopeptides.^12^ We have exploited multicomponent cavitand : dye arrays for the detection and discrimination of a variety of biological targets with miniscule differences in structure,^13^ including oligonucleotides,^14*a*,*b*^ drugs of abuse^14*c*^ and post-translationally modified peptides.^14*d*–*f*^ Here we extend that work, and show that a combination of water-soluble synthetic hosts and cationic fluorophores can selectively recognize isomerization of single residues in oligopeptide strands, exploiting an indirect, competitive recognition mechanism.

**Fig. 1:**
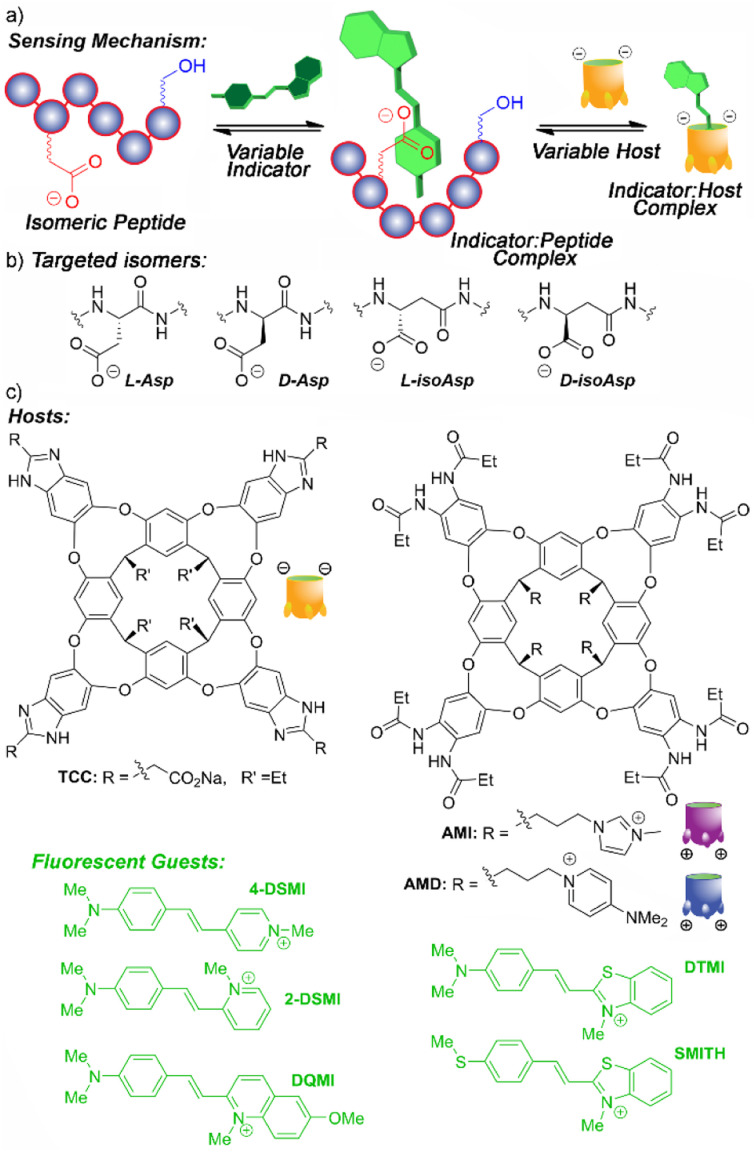
Illustrations of (a) the peptide isomer recognition and sensing mechanism; (b) isomer variants of aspartate; (c) hosts and indicator dyes used in this study.

Of course, the challenge in detecting small changes in peptide structure with synthetic host molecules and associated indicators is that there is no obvious “binding handle” for the receptor to target. Hosts such as TCC (Fig. 1) are selective for soft cations such as R-NMe_3_^+^,^14*d*,15^ so are highly effective at recognizing trimethyllysine modifications through a simple indicator displacement assay.^14*d*,*e*^ Isomerization and epimerization of individual residues confers no change in the atomic constituents of the target, just the 3D structure, so at first glance may seem unsuited to this technique. However, one of the advantages of deep, water-soluble cavitands such as TCC is their ability to exploit multiple different types of recognition mechanism,^13,14^ which allows for a wide scope of targets that can be sensed.

Instead of using the host to bind a specific molecular motif (*e.g.* Kme_3_ (ref. 15)), and directly displacing a bound indicator dye, the dye itself can be used as the recognition element.^14*a*^ In this case, differential sensing can be employed by using a competitive recognition process whereby the host and target both bind the dye. This sets up competing equilibria in the system, and the relative binding affinities of host·dye and target·dye control the fluorescence output of the array. Employing variable hosts and dyes with different affinities for each other and for the target adds an extra layer of differentiation for small differences in target structure. We have previously used this concept to detect changes in the structure of non-canonically folded DNA. Dyes such as DSMI (Fig. 1) can bind to folded DNA, and the introduction of hosts can provide a competitive recognition system, modulating the dye emission and allowing discrimination of small changes in structure.^14*a*,*b*^ A slightly different, yet related concept has been used to sense phosphorylation of unmodified peptides using hosts such as TCC and DSMI dyes: in this case, the host interacts with the cationic, hydrophobic peptide and is repelled by the introduction of phosphorylation.^14*f*^

These indirect sensing methods allow different strategies for creating a recognition system: either the dye or the host can act as the “recognition element”, and the presence of the other partner allows modulation of affinity, providing variables for differential sensing. The challenge for detecting peptide isomers is that there are few reliable recognition motifs for small molecules in unmodified oligopeptide strands. The phosphorylation detection^14*f*^ was successful for highly cationic peptides, as they interacted with the anionic TCC. As oligopeptides show a vast array of different properties including charge, lipophilicity, presence of aromatic π-stacking groups, a “one-size-fits-all” recognition system is not realistic. However, as deep cavitand hosts and styrylpyridinium dyes are quite promiscuous in their recognition abilities, we were interested in whether they could be exploited for detection of novel peptide target structures *via* as yet unknown mechanisms. As such, we performed an initial screen of a series of styrylpyridinium dyes (Fig. 1) that have been shown to be good guests for TCC,^13,14^ with a series of peptide strands. This preliminary screen should hopefully show that specific peptide sequences can interact with the dyes, and are amenable to detection and discrimination.

## Results and discussion

The initial peptide targets consisted of three groups of peptides from disease-related, long-lived proteins: αB-crystallin 57–69,^5*c*,9*c*^ Aβ 1–10,^5*c*^ and human tau 382–395,^9*d*,16^ as well as four peptides from histone H3 as controls, peptides that we have used previously in cavitand-based sensing^14*d*,*e*^ (Table 1). These peptides cover a wide range of pI and hydrophobicity values while retaining similar sizes. For the αB, Aβ and tau peptide strands, two synthetic peptide variants were tested containing either L-Asp or D-Asp at the relevant residue (see ESI†). Each of these 10 peptides (4 μM) was initially added to a solution of one of the five dyes (0.5 μM), in 20 mM Tris buffer, pH 7.4.

**Table 1:** Peptide Strands Tested^a^

Name	Sequence^b^	pI	Charge^c^	Hydro
Tau (382–395)	AKAKT*D*HGAEIVYK	9.56	+1	14.49
Aβ (1–10)	DAEFRH*D*SGY	4.29	−2	13.14
αB (57–69)	APSWF*D*TGLSEMR	4.09	−1	33.09
H3 (1–21)	ARTKQTARKS	12.71	+7	2.72
	TGGKAPRKQLA			
H3 (1–11)	ARTKQTARKST	12.41	+4	−0.29
H3 (23–34)	KAARKSAPATGG	11.65	+3	3.56
H3 (73–83)	EIAQDFKTDLR	4.31	−1	25.19

a See Table S-1 for additional peptide properties.

b Multiple isomers tested: residue labeled in bold is the site of isomer variation.

c Net charge at pH 7.4.

**Fig. 2:**
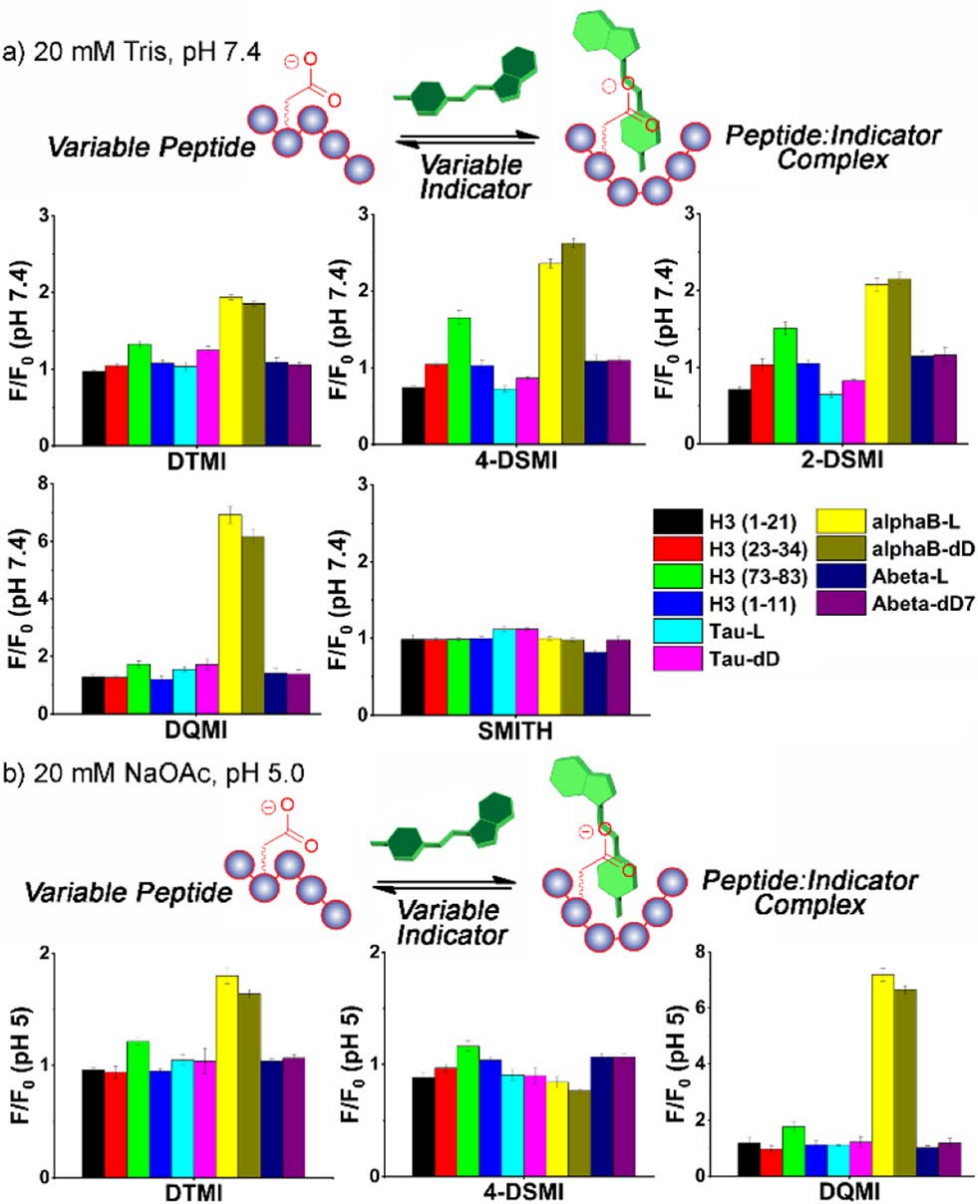
Fluorescence response plots of various dyes added to various peptide strands in (a) 20 mM Tris-HCl buffer, pH 7.4 or (b) 20 mM NaOAc buffer, pH 5. [dye] = 0.5 μM, [peptide] = 4 μM. *F*_0_ = fluorescence response in the absence of peptide.

**Fig. 3:**
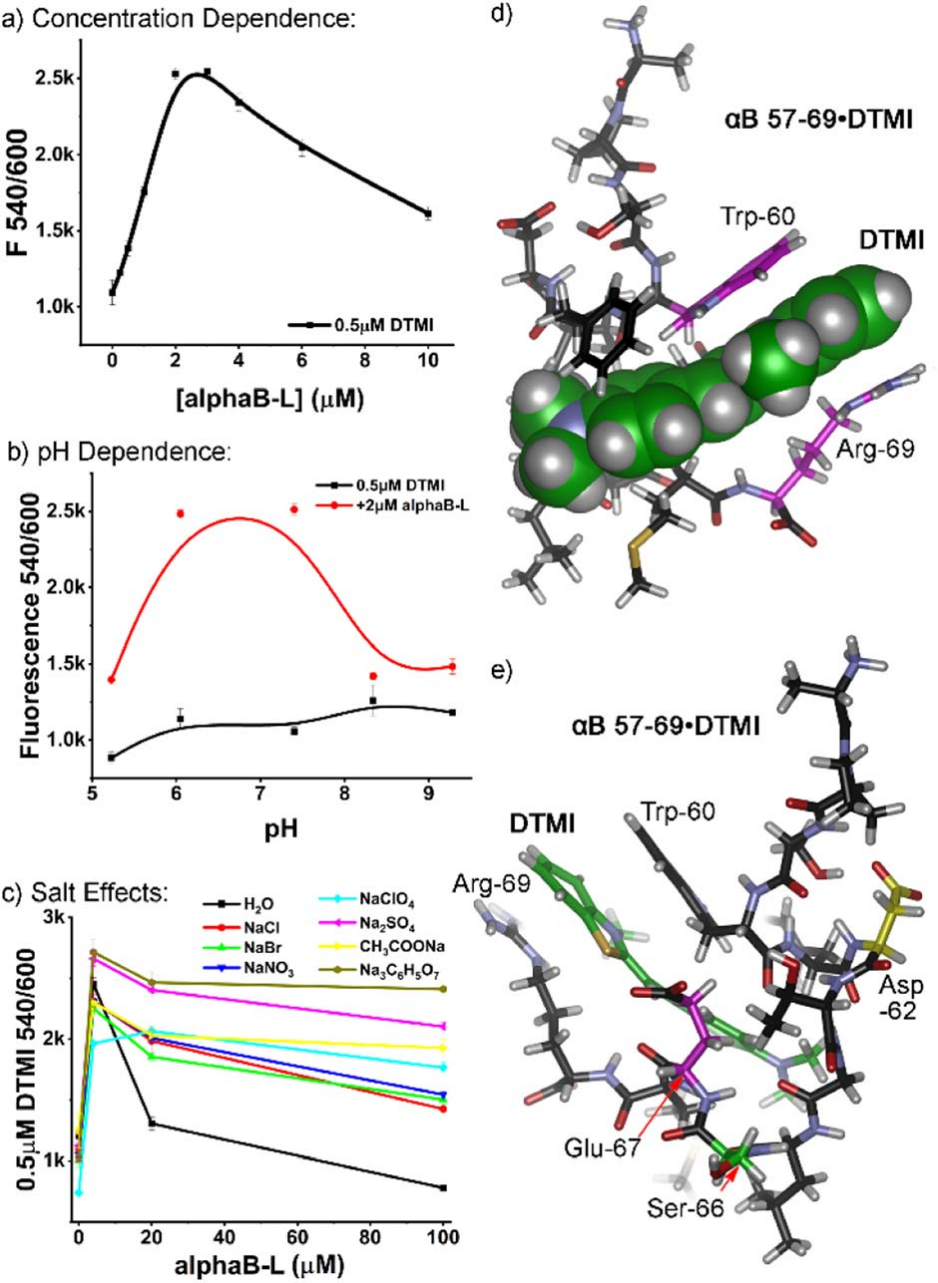
External additive effects on the fluorescence behavior of DTMI in the presence of αB 57–69. (a) DTMI emission (0.5 μM) dependence on [αB 57–69] in water; (b) comparison of DTMI emission in the presence and absence of αB 57–69 at various pH; (c) effect of 500 mM salt on the DTMI emission with increasing [αB 57–69] in water. (d and e) Different views of the optimized structure of DTMI•[αB 57–69] modeled by molecular dynamics simulations.

**Fig. 4:**
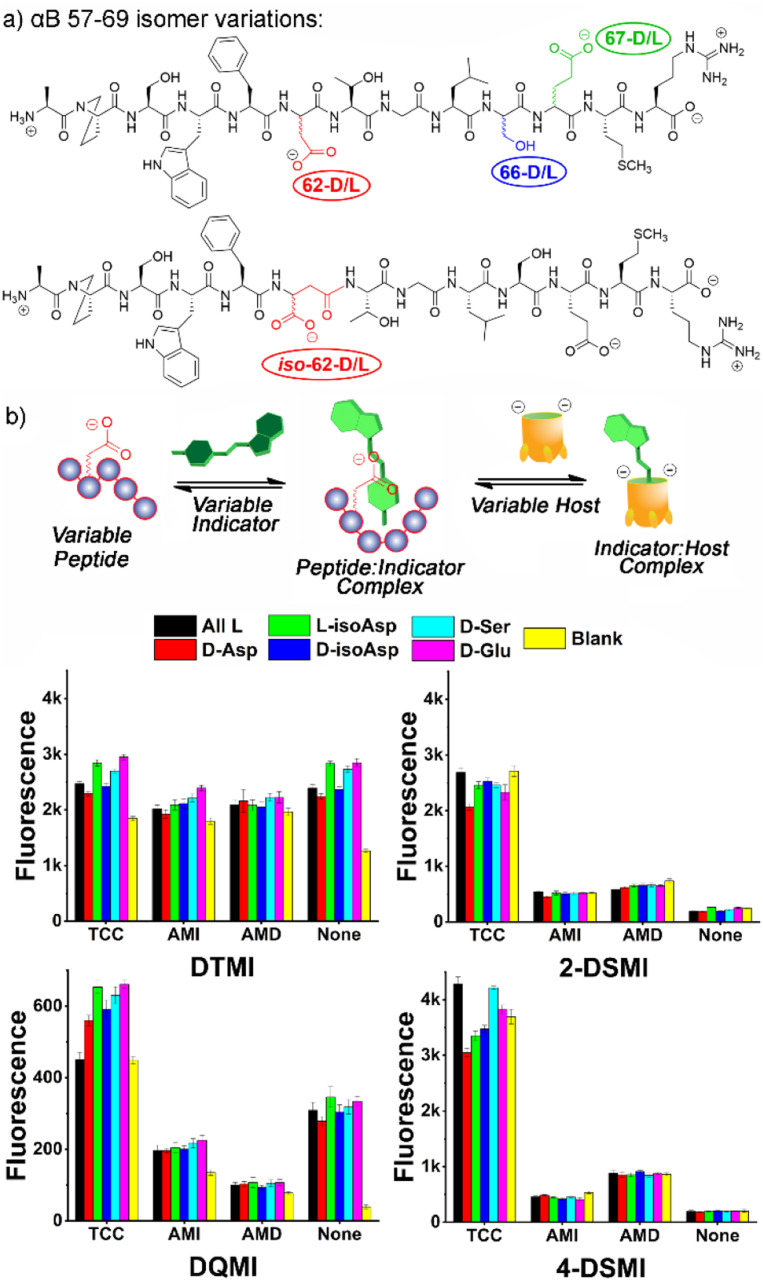
(a) Isomeric variations in αB 57–69 tested; (b) fluorescence response plots of cavitand : dye combinations added to 6 alphaB isomers. [Dye] = 0.5 μM, [Host] = 1 μM, [peptide] = 4 μM, in 20 mM NaOAc buffer at pH 5.0.

**Fig. 5:**
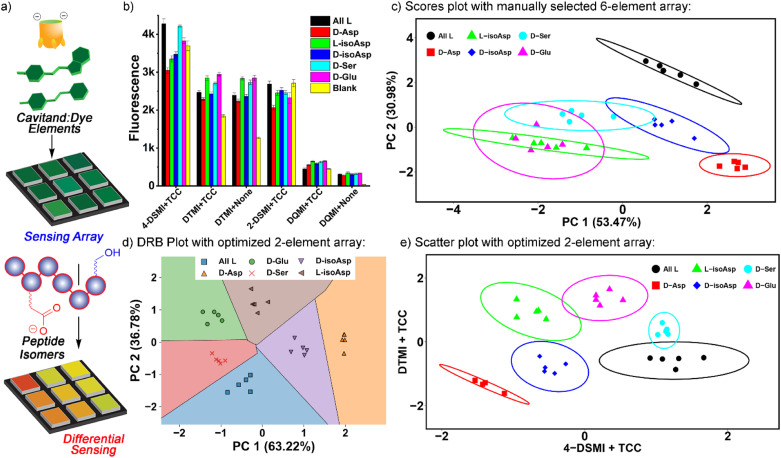
(a) Differential sensing concept. (b) Fluorescence emission data for the classification of 6 alphaB isomers with a manually chosen 6-element array: TCC•4-DSMI, TCC•DTMI, DTMI, TCC•2-DSMI, TCC•DQMI, and DQMI, in NaOAc pH 5 buffer. [Dye] = 0.5 μM, [Host] = 1 μM, [peptide] = 4 μM. (c) PCA scores plot of the emission data shown in part (b). (d) Decision region boundary plot for isomer discrimination using the PCA of the 2 most optimal elements selected by SVM-RFECV: TCC•4-DSMI and TCC•DTMI. (e) Scatter plot for isomer discrimination using the 2 most optimal elements selected by SVM-RFECV using the scaled *F*/*F*_0_ values.

The initial screen was quite surprising – as can be seen in Fig. 2a and S-5 – S-9,† both epimers of αB-crystallin 57–69 (hereinafter referred to as αB 57–69) effected a significant increase in fluorescence (between 2- and 6-fold) for four of the dyes in the screen, **DTMI**, **2-DSMI**, **4-DSMI** and DQMI, at a concentration of only 4 μM peptide. This increase was quite specific for αB 57–69: neither Aβ 1–10 nor Tau 382–395 gave any signal change at all, nor did any of the H3 peptides except H3 (73–83). The effect was also quite dependent on dye structure, as SMITH, the thioether variant of DTMI, showed no emission increase for any of the peptides. Furthermore, a small but discrete difference in the emission of the 4 successful dyes was seen in the presence of different isomers of αB 57–69 (D-Asp62-αB and L-Asp62-αB).

These results indicate that these cationic, hydrophobic dyes associate with αB 57–69, causing an emission increase, but have minimal response to the other peptides. This was unexpected, so we performed further tests to determine the mechanistic underpinnings of the recognition. When the screen was repeated at pH 5 (20 mM NaOAc buffer, Fig. 2b – see ESI† for full plots), the picture became more complex. The interactions of the dyes at pH 5 with the peptides were not consistent with the effect at pH 7.4. While there were no changes in the “unsuccessful” pairings (*i.e.* peptide : dye combinations that showed no emission enhancement at pH 7.4 also showed no enhancement at pH 5), certain dyes, namely 2-DSMI and 4-DSMI, ceased to show any enhancement with αB 57–69 at the tested concentrations. Despite the similarity in structure of the different dyes, significant differences in recognition for peptides were observed upon minor changes in external conditions.

While the selectivity of the dyes for the peptides was variable, there were consistent properties in the peptides that showed response. Cationic peptides (high pI, *e.g.* H3 (1–21)) showed no response, which is unsurprising, given that the dyes have a constant positive charge. The effect was not purely charge-based, though – Aβ 1–10 showed no effect on dye properties, despite its low pI. The other property that appeared essential was hydrophobicity. Calculated values of peptide hydrophobicity (Table 1) and GRAVY (see Table S-1†) show that a combination of favorable charge matching (low pI, net negative charge) and high hydrophobicity favors dye binding and emission enhancement. The two peptide strands that share these properties, αB 57–69 and H3 (73–83), both show indicator response. It should be noted that DTMI is quite similar in structure to thioflavin T (ThT), a common dye for sensing peptide aggregates,^17^ so the affinity for hydrophobic residues is consistent.

Further experiments were performed to identify the most favorable conditions for peptide : dye interaction, focusing on αB 57–69. The similarity in the structure between the screened dyes and ThT suggested that peptide aggregation may be *a* factor, so we analyzed the effects of peptide concentration on the emission profiles. Increasing concentrations of αB 57–69 were added to 0.5 μM dye in ultrapure water. The profiles varied depending on the nature of the dye, but as can be seen in Fig. 3a for DTMI (see Fig. S-10 for full data†), increasing peptide concentration causes an initial spike in emission (up to 2.3-fold in this case) from 0–2 μM peptide, followed by a plateau, and then a drop in emission as [peptide] increases further. However, at these low concentrations, it is highly unlikely that peptide oligomerization into larger superstructures occurs, and no attempts were made to pre-aggregate the peptides into sheets: they were added fresh, with no incubation time.^18^ These results indicate that the response is *not* seen with extensively aggregated peptides (an observation also supported by the lack of response for Aβ 1–10). Obviously some change in supramolecular structure is occurring, though, and simple dimerization or formation of small aggregates cannot be ruled out.

If the peptides are aggregating somewhat, this would be affected by external constituents, so we investigated the effect of pH in more detail. It became immediately clear that the dyes have a window of suitability: at pH > 9, the emission enhancement of the dyes with αB 57–69 was abrogated, and in some cases (DTMI), the dye emission itself irreversibly dropped, indicating decomposition. At low pH (3 or lower), some dyes were effective, but the relative enhancement dropped when compared to the “sweet spot” of pH 5–7 (see Fig. 3b, S-11 and S-12†).

Finally, we investigated the effects of added Hofmeister salt on the emission. The titration of αB 57–69 into a 0.5 μM solution of DTMI in H_2_O was repeated in the presence of 500 mM of various Hofmeister salts.^19^ Salts at the chaotropic (perchlorate), kosmotropic (citrate, sulfate, acetate) and center (halides, nitrate) of the Hofmeister series were tested (Fig. 3c, S-13†), and the pH was maintained at neutral by adding small amounts of HCl or NaOH after the sample had been prepared. Interestingly, the nature of the added Hofmeister salt had a minimal effect on the initial emission enhancement of DTMI with 4 μM peptide: the enhancement only varied from ∼2-fold to 2.5-fold. However, the effect on the decrease in emission as the [peptide] increased was stark: while the emission enhancement was completely abrogated by 100 μM peptide in ultrapure water, the presence of salts prevented this decrease, with the nature of the salt affecting the amount of prevention. While there is not a completely consistent trend between the salting-out or salting-in nature of the salt and the effect on emission, the kosmotropes (citrate, sulfate, acetate) almost completely prevented the drop in emission of DTMI at higher [peptide], up to 100 μM. All other salts reduced the emission drop compared to water, albeit to a lesser degree.

As the nature of the peptide affinity for the dyes was complex, we turned to theory to determine a plausible interaction mechanism. Molecular Dynamics (MD) simulations were performed for the association of DTMI and αB 57–69 in a water box using the AMBER20 simulation package with ff14sb and GAFF2 force fields for the peptide and dye, respectively.^20^ We post-analyzed trajectories of 500 ns MD runs and calculated interaction energies between the dye and various conformations of αB 57–69 using the molecular mechanics/Poisson-Boltzmann surface area (MM/PBSA) method. MD captured the ensemble of the complex conformations, where DTMI was bound to the peptide with folded (Fig. 3d and e), partially folded, and extended peptide conformations (see Fig. S-4† for minor conformation images).

Fig. 3d and e display the most favorable structure of the DTMI•αB 57–69, where the complex holds the strongest interaction energy and longest association time. The peptide folds into a cleft-like structure that maximizes the facial π–π interactions between the Trp-60 and Arg-69 residues and the cationic, conjugated DTMI. The other next-lowest energy conformations sampled by MD all involved the Trp-60 residue in the interaction with DTMI, but this particular conformation was most favorable. It must be stressed that the dye could show affinity for other conformations of the peptide: similar interactions could easily be observed between dyes and other transiently folded conformations, and Phe-61 is also a sidechain candidate for π-stacking with the dye. However, the optimized structure does provide an explanation for the emission enhancement with DTMI and related dyes. We have previously shown the emission of these dyes is enhanced in the presence of aromatic stacking interactions, either with cavitands and/or G-quadruplex DNA;^14^ selectivity for Trp-rich peptides is consistent with this. The folded structure also provides a glimpse of the possible selectivity for sensing isomeric variations in the peptide structure: our initial tests used the D- and L-epimers of αB 57–69 at Asp-62 (Fig. 2), highlighted in yellow in Fig. 3e. This residue is close to the binding “pocket”, and so epimerization would change the peptide structure and could concomitantly change the affinity of the dye for the peptide. The fluid nature of the binding pocket also explains the high degree of variation in emission for different types of dye, as well as the effect of pH on the emission. Small changes in dye structure cause changes in “fit” in the pocket, hence changes in emission, and the intimate interaction between dye and charged residues will also have an effect.

However, this monomeric structure does not completely explain the drop in emission at high [peptide]. The emission titration data exhibits a biphasic response relationship with peptide concentration. This is reminiscent of the low-dose stimulation and high-dose inhibition phenomena observed with two receptor subtypes.^21^ Therefore, we performed a Hill1 fitting for the curves in two regimes separately, either increasing (mimicking stimulation) or decreasing (mimicking inhibition) fluorescence. The *k* and *n* values in the Hill1 equation originally represent the half-maximal concentration constant and number of cooperative binding sites of binding. While only an approximation, these values can hint at the dye : peptide binding affinity and stoichiometry, respectively. For DTMI and αB 57–69, a low micromolar affinity (*k* ∼1 and 6 μM, respectively, for the “stimulation” and “inhibition” regime) was observed (see Table S-3† for full data), suggesting relatively strong binding between the dye and the peptide or small peptide aggregates. The *k* values for the fluorescence decreasing curve are 3–10 times larger than those for the fluorescence increasing curve, suggesting lower affinity in the “quenching” regime. As such, a theory can be postulated that formation of small dimers or oligomers of peptides in solution at higher concentration causes expulsion of the dye from the peptide, corroborated by the lower affinity seen at higher [peptide]. Alternatively, changes in the stoichiometry of binding could cause self-quenching of the dye,^14*d*^ but this seems less likely. Why the addition of kosmotropes (“salting-out” anions) should have the greatest effect on the aggregation is not clear, but the prevention of signal loss by anion addition does not follow Hofmeister trends (chaotropes are also effective, but halides are not), so any theory here is premature. Ion effects on aggregation are complex,^22^ so more mechanistic detail would be needed.

While the peptide : dye binding events are quite complex, this data leads to a simple conclusion: the four different dyes can bind the αB 57–69 peptides with varying affinity and variable emission. This provides the impetus for selective detection and discrimination of small structural changes such as peptide isomers. The changes in dye emission are small, but perfectly suited for differential sensing. However, a second element is desirable to enhance selectivity: this is where deep cavitands are invaluable.^13^ We had previously shown that the TCC cavitand has a strong (sub-micromolar) affinity for *cationic* peptides such as H3 (1–21) at pH 7.4.^14*f*^ This was attributed to favorable matching of charge and hydrophobicity between the anionic, lipophilic host and the cationic (pI 12.71) peptide. This sets up the possibility that hosts such as TCC can compete for recognition, either for the dyes, for the peptide, or both, forming heteroternary complexes.^14*a*,*b*^ It was not clear what the affinity of TCC for the anionic αB 57-69 would be, so we also tested other cationic hosts AMI and AMD, which have previously been used to sense structural variations in DNA.^14*a*,*b*^

At this point, we also focused on the central goal, namely sensing structural changes in the peptides from introduction of isomeric residues. Six peptides were tested, the core all-L αB 57-69 peptide and 5 isomeric variants: epimers of αB 57–69 at Asp 62, Ser 66 and Glu 67, as well as the D/L isomers of αB 57-69 with iso-aspartate at residue 62 (see Fig. 4a for structures and Table S-2† for the location and type of each isomeric amino acid in the sequence). These peptides (4 μM) were added to solutions of one of the 4 previously successful dyes (2-DSMI, 4-DSMI, DTMI, DQMI) in 20 mM NaOAc buffer at pH 5.0, in the presence (or absence) of 1.0 μM cavitand, either TCC, AMI or AMD (Fig. S-19 and S-20†). This buffer was chosen for two reasons – a constant pH of 5.0 could be maintained easily, and acetate showed good protection of the emission signal at higher peptide concentrations.

To determine the most effective sensor array for differentiation, we applied two strategies: simple manual inspection of the fluorescence data, followed by application of a machine learning algorithm to identify the most effective elements. The manual elimination step was quite simple – which element caused the most obvious differences in signal for the 6 different αB 57-69 isomers in Fig. 3? The choice of dyes was simple, as DTMI, 2-DSMI, 4-DSMI and DQMI all show variable enhancement upon addition of the peptides, whereas SMITH is unchanged, so was removed. The two cationic cavitands AMI and AMD did cause some changes in signal, but they were minor, and in some cases negligible (Fig. 4b), so TCC was chosen as the sole cavitand additive. This leaves 8 potential elements: the 4 dyes alone, and each dye + TCC. As the emission of the dyes was somewhat affected by salts, we also tested some additives such as the salting-in anion ClO_4_^−^ (see ESI† for full data), but this showed no improvement in peptide differentiation (Fig. S-18†), so was eliminated from the array. Finally, pH is a possible variable, but as described earlier, the largest differences in emission from the TCC•dye complexes in the presence of peptides are seen in 20 mM NaOAc buffer at pH 5.0, so for simplicity and consistency, we chose that pH for optimization. This causes one more reduction: while 2-DSMI and 4-DSMI show a strong enhancement at pH 5.0 in the presence of TCC and peptides (Fig. 4b), the change in signal for the dyes alone is very small, so these two dyes in the absence of TCC were excluded.

This leaves six obvious potential sensor elements that could be used to construct a positionally- and residue-selective differential sensor for αB-crystallin peptide isomers. To quantitate the differentiation effect of these sensors, we calculated the ratio (*F*/*F*_0_) of the sensor fluorescence collected with (*F*) or without (*F*_0_) the peptide (Fig. 5b), and subjected the ratios of all six sensors, *i.e.*TCC•2-DSMI, TCC•4-DSMI, TCC•DTMI, TCC•DQMI, DTMI and DQMI, to Principal Component Analysis (PCA). Disappointingly, the separation of the six elements was quite poor using the full 6-element array (Fig. 5c). While full discrimination was possible for some elements (for example, all four of the variants at Asp-62 were fully separated and no overlap was seen with the 95% confidence ellipses), the discrimination was far less successful for the epimers at Ser 66 and Glu 67. This illustrates the limits of manual choice of elements for differential sensing: despite the differences in emission that can be observed from a simple visual inspection, not all elements are effective at discriminating all the peptides, and this is difficult to spot upon simply looking at the emission. This also reinforces an important point about differential sensing: adding more elements does not necessarily improve the discrimination, as deleterious elements often reduce the selectivity.^13,14*b*^

To identify the most optimal sensor elements, we treated the scaled *F*/*F*_0_ data with the SVM (support vector machine)-RFECV (recursive feature elimination with cross-validation) machine learning algorithm,^23^ which can select the most informative features for designated sample classification among all those used to generate the database (Fig. S-22†). Setting the classification goal as differentiating all six αB 57–69 isomeric variants, SVM-RFECV found that only two sensor elements, TCC•4-DSMI, TCC•DTMI, are needed to classify all six peptides (see Table S-4†), resulting in ideal (=1.00) average (“macro”) scores of accuracy, sensitivity, specificity, precision, and AUC from 3 repeated 4-fold cross validation tests (see Table S-5†). The results of this contraction can be seen on the SVM decision boundary plot (Fig. 5d), which illustrates good separation of all 6 isomers by the simple two-component array. Since only two sensor elements are required for successful classification of these peptide variants, directly projecting the scaled fluorescence ratios attained from these two elements in a 2-D scatter plot (as opposed to the PCA scores plot shown in Fig. S-25†) should also provide good differentiation. Indeed, as can be seen in Fig. 5e, this simple 2-element array can almost completely differentiate all 6 of the peptides from each other: all five repeats for each peptide are clustered closely, and the different peptides are well separated with minimal overlap of their 95% error ellipses (Fig. 5e). The only overlap is between D-Ser 66 and the unmodified αB 57–69, as well as a small overlap between D-Glu 67 and L-isoAsp 62. The challenge in detecting the smallest residue isomer (D-Ser 66) is to be expected, and it is impressive that this isomer can be discriminated at all. The scatter plot also illustrates the power of individual elements in isomer discrimination. While both elements are needed to discriminate all six isomers, TCC•4-DSMI can differentiate the peptide variants into broad classes, with or without the isomeric Asp 62. On the other hand, TCC•DTMI can successfully separate the D/L isomers of Asp 62 and iso-Asp 62, while also showing positional selectivity for epimerization at Ser 66 and Glu 67.

**Fig. 6:**
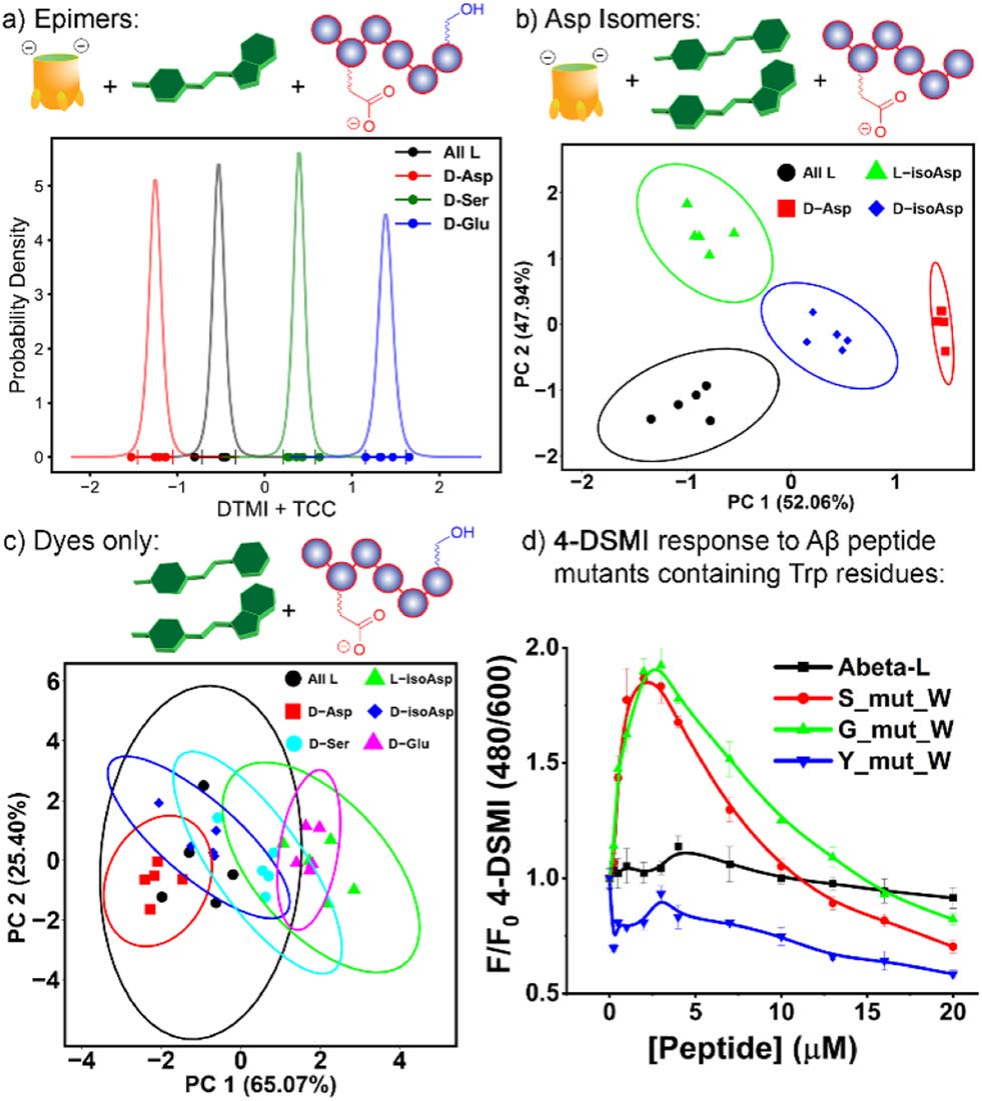
(a) *t*-distribution plot for differentiation of four αB 57–69 epimers using one sensing element, TCC•DTMI; (b) PCA scores plot for discrimination of four Asp-62 isomers using two sensing elements, TCC•4-DSMI/TCC•DTMI. The fluorescence responses used in data analysis are included in Fig. 5b. Ellipses at 95% confidence interval; (c) PCA scores plot indicating the ineffective discrimination of the six peptide isomers with an array consisting of DTMI, 2-DSMI, 4-DSMI and DQMI in the absence of cavitand; (d) 4-DSMI emission (0.5 μM) dependence on [Aβ (1–10)] mutants containing Trp residues in H_2_O.

The excellent performance of the 2-element TCC•4-DSMI/TCC•DTMI array at discriminating all 6 isomers with only minor overlap suggested that by narrowing the target scope, even more robust discrimination would be possible. As such, we focused on two sets of 4 isomers: the epimerization isomers of αB 57-69, comparing the parent peptide with D-Asp 62, D-Ser 66 and D-Glu 67, and the four variants at Asp 62 (D/L and isoAsp). As can be seen in Fig. 6 (and Fig. S-27–S-31†), simple array elements are capable of fully discriminating these two sets of isomers. For the epimers (Fig. 6a), only one element is required: simple application of TCC•DTMI enables full separation on a 1D *t*-distribution plot. Discrimination of the various Asp-62 isomers is slightly more challenging, but even then, only (TCC•4-DSMI/TCC•DTMI) are required. As can be seen in Fig. 6b, no overlap between the various repeats on the PCA scores plot is seen, so complete discrimination is possible with a simple, minimal array.

Of course, this minimal array could suggest that the dyes alone were able to discriminate the peptide isomers, but this is not the case. When the emission data for an array formed from the four “best” dyes DTMI, 2-DSMI, 4-DSMI and DQMI was analyzed by PCA for either the full 6-isomer target set or the smaller 4-isomer set described above, no discrimination was observed at all (Fig. 6c and ESI Fig. S-32–S-33†). This highlights the importance of the cavitands in the array: the dyes themselves can recognize the peptide strand and form inclusion complexes, but the affinity for the different dyes and the isomeric peptides varies so little that they are not competent to differentiate small changes in structure, which is to be expected. The dyes are all quite similar in structure, and small changes in flexible oligopeptide structures as at single residue do not have a large effect on the ability of the peptide to fold around the target. The introduction of the cavitand(s) amplifies those differences, however – when small differences between host•dye binding are paired with small differences in dye·peptide binding, those differences are magnified to the extent that complete differentiation is possible. Some combinations are not effective, but when machine learning is applied to detect the most important array elements, good discrimination is possible even when using a small array.

Fig. 3d and e suggest that the Trp-60 residue in the flexible oligopeptide is an important factor that confers affinity for the peptides to the dyes, and fluorescence enhancement upon binding. This provides an easily detectable output signal for peptide recognition, and is also the base of isomer differentiation by our host·dye sensor array. To illustrate the scope of possibilities for detection of structural variations in more peptides, we tested whether introduction of Trp residues to other peptides would confer a fluorescence response. Three mutants of Aβ (1–10) were synthesized, with Trp residues at varying positions – S8W, G9W and Y10W (see Fig. 6d, ESI S-34 and S-35 for data and Table S-10† for full peptide sequences). The wild-type sequence of Aβ (1–10) does not contain W, nor does it induce fluorescence responses in the dyes tested (Fig. 2a and b). The results with the W mutants were encouraging, and mirrored the response of αB 57-69: whereas neither 4-DSMI nor DTMI showed any increase in emission upon addition of the wild-type Aβ (1–10), titration of Aβ(1–10)S8W and Aβ(1–10)G9W caused a significant increase in the emission (up to 2-fold) of both 4-DSMI and DTMI. Interestingly, the dye response curves for the different dyes behaved differently – the 4-DSMI response to Aβ(1–10)S8W and Aβ(1–10)G9W was very similar to that shown to αB 57–69, with an initial spike, followed by a loss of signal at high [peptide]. In contrast, the DTMI response to Aβ(1–10)S8W and Aβ(1–10)G9W was similar to standard saturation binding. Also, introduction of Trp at the peptide terminus was ineffective, as the response of both 4-DSMI and DTMI to Aβ(1–10)Y10W was negligible, similar to that of the wild-type. This is presumably due to the added flexibility of the side-chain at the peptide terminus causing an entropic penalty to folding and forming a binding “pocket”, thus lowering the affinity for the dyes. However, this data illustrates the possibility of the sensing system: for flexible oligopeptides containing electron-rich aromatic residues in the oligopeptide interior, recognition is possible with cationic aromatic indicators. This recognition can be leveraged to detection of small changes in peptide structure – in this case, we have shown the detection of peptide isomers, but this recognition mechanism could also conceivably be used to detect other modifications such as lysine acylation, serine phosphorylation, among others.

## Conclusions

Here, we have shown that a simple set of cationic dyes can bind selected peptides with variable, yet strong affinity, which allows them to be used to detect minute changes in peptide structure such as single residue isomerization. Molecular dynamics simulations allow the determination of the most favorable peptide : indicator conjugate structure, and suggests that the recognition system is most effective for hydrophobic peptides, and is aided by the presence of tryptophan residues in the backbone, which allow π-stacking with the cationic dyes. The scope of the recognition can be extended to other peptides by incorporating W mutants in the backbone. By pairing this recognition with competitive binding with a water-soluble deep cavitand, a differential array can be created that allows complete discrimination of single isomeric Asp, Glu or Ser residues in αB-crystallin peptides. Machine learning optimization can reduce array dimensions to only two elements, so there is no complex post-detection processing necessary, and the recognition system is fully functional in biorelevant media. This demonstrates the power of the detection system in achieving subtle discrimination of highly similar structures. Peptide isomers are rarely targeted for selective optical detection despite their importance in diseases related to long-lived proteins, so we believe this type of array-based sensor will enable further study of these systems.

## Data availability

Raw experimental and computational data is available upon request to the corresponding author.

## Author contributions

RJH, WZ and JC conceived of the project and wrote the manuscript, with assistance from C-EAC, PF and JM. JM, BLH and AAPR performed chemical synthesis of cavitands and dyes, JC performed the sensing experiments and PF and C-EAC performed the theoretical calculations.

## Conflicts of interest

There are no conflicts to declare.

## Supplementary Material

SC-015-D3SC06087J-s001
