# Supplementary material for: Selective recognition and discrimination of single isomeric changes in peptide strands with a host : guest sensing array
Source: Chem Sci. 2024 Jan 2;15(5):1885–93. doi: 10.1039/d3sc06087j (PMC10829040; doi:10.1039/d3sc06087j)
Supplement: SC-015-D3SC06087J-s001 [file SC-015-D3SC06087J-s001.pdf]

Electronic Supplementary Material (ESI) for Chemical Science.
This journal is © The Royal Society of Chemistry 2024

Selective Recognition and Discrimination of Single Isomeric Changes in Peptide Strands with a Host:Guest Sensing Array

Junyi Chen,[b] Parisa Fasihianifard,[a] Alexie Andrea P. Raz,[a] Briana L. Hickey,[a] Jose L. Moreno, Jr.,[a] Chia-En A. Chang,[a] Richard J. Hooley[a,b]* and Wenwan Zhong[a,b]*

[a]*Department of Chemistry;* [b]*Environmental Toxicology Graduate Program; University of California–Riverside, Riverside, CA 92521, U.S.A.*

E-mail: richard.hooley@ucr.edu; wenwan.zhong@ucr.edu

Electronic Supplementary Information

Table of Contents

1. General Information……………………………………………………………….……………..*S-2*

1.1 Chemical Synthesis and Characterization……………………………………..*S-3*

1.2 Fluorescence Measurements……………………………………………………*S-3*

1.3 Molecular Dynamics Simulations and Interaction Energy Calculations……….*S-5*

1.4 Data Analysis……………………………………………………………………*S-6*

2. NMR Characterization of New Molecules…………………………………………*S-7*

3. Peptide Sequences and Asp Isomerization…………………………………………...*S-8*

4. Molecular Dynamics Simulation……………………………………………………..*S*-10

5. Dye-Peptide Interaction………………….………………..…….…………….…….....*S-11*

5.1 Impacts on Dye (DTMI, DQMI, 4-DSMI, 2-DSMI and SMITH) Fluorescence by Various Peptides in pH 7.4/5 Buffers……………………………………………..*S-11*

5.2 Impacts on Dye (DTMI, DQMI, 2-DSMI, 4-DSMI, SMITH, ThT) Fluorescence by Peptide Concentration……………………………………………………………..*S-16*

5.3 Effects from pH on Dye (DTMI, DQMI, and 4-DSMI) Fluorescence…………*S-18*

5.4 Effects from Salts on Dye (DTMI, DQMI, and 4-DSMI) Fluorescence ……….*S-20*

6. Array Sensing of AlphaB Isomers…………………………………………………...*S-21*

6.1 Host•Dye (DTMI/4-DSMI/2-DSMI) Sensors at pH 7.4……….……………….*S-21*

6.2 The 12-Element Array with Added Salts………………………………………..*S-23*

6.3 The 16-Element Array at pH 5…………………………………………………..*S-25*

6.4 Dye Titration of Trp (W)-Containing Abeta (Aβ) Mutant Peptides…………..*S-39*

7. References……….………………………………………………………..………..*S-41*

1. General Information

Cavitands **TCC**,[1] **AMI**,[2] **AMD**[3] and fluorophores **4-DSMI**,[4] **2-DSMI**,[3] **DQMI**[5] and **DTMI**[3] were synthesized and characterized according to literature procedures. The dye stock solutions were prepared in DMSO (Fisher Chemical, Catalog Number: D128-1) at a concentration of 20 mM, and later diluted with water for use in experiments. The pyrogen-, nuclease- and bacteria-free ultrapure (Type 1) water produced by Direct-Q 3 UV water purification system with Biopak polisher (Catalog Number. CDUFBI001), was used in peptide experiments. Peptides with HPLC purification were purchased from Biomatik, the sequence modification of which are given in Table *S*-1. The concentrations of peptide stock solutions were determined by NanoDrop 2000 (Thermo Fisher Scientific) using the corresponding molar extinction coefficients at 280 nm in water calculated by Expasy ProtParam tool (https://web.expasy.org/protparam/) after background subtraction. The pH values of solutions were measured by Fisher Scientific Accumet Excel XL50 Dual Channel pH/Ion/Conductivity Meter with Fisherbrand Accumet glass body standard size combination electrode (Catalog Number 13-620-223A), which was pre-calibrated using RICCA Chemical pH buffer reference standard solutions of pH 4.00, 7.00, and 10.00 (Catalog Number 1501-16, 1551-16, and 1601-16). Solvents were dried through a commercial solvent purification system (Pure Process Technologies, Inc.). All other chemical were purchased from Sigma-Aldrich, Alfa Aesar, TCI or Fisher Chemicals and were used as received. ^{1}H and ^{13}C NMR spectra were recorded on Bruker Avance NEO 400 MHz and 600 MHz NMR spectrometer. The spectrometers were automatically tuned and matched to the correct operating frequencies. Proton (^{1}H) and carbon (^{13}C) chemical shifts are reported in parts per million (δ) with respect to tetramethylsilane (TMS, $\delta = 0$), and referenced internally with respect to the protio solvent impurity. Deuterated NMR solvents were obtained from Cambridge Isotope Laboratories, Inc., Andover, MA, and used without purification. High resolution accurate mass spectral data were obtained from the Analytical Chemistry Instrumentation Facility at the University of California, Riverside, on an Agilent 6545 QTOF LC/MS instrument.

1.1 Chemical Synthesis and Characterization

Synthesis of (E)-3-methyl-2-(4-(methylthio)styryl)benzo[d]thiazol-3-ium iodide (SMITH):

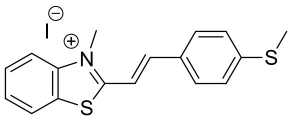

2-methylbenzothiazole (200µL, 1.60 mmol) was dissolved in ethanol (5 mL), iodomethane (1 mL) was added to the reaction mixture while stirring and the reaction was refluxed for 12 hours. The solution was diluted with diethyl ether (10 mL) and the resulting precipitate was filtered, then rinsed with diethyl ether and dried under vacuum to yield 2,3-dimethylbenzothiazol-3-ium iodide (398 mg, 87%) as a white solid, which was used directly in the next step. 2,3-dimethylbenzothiazol-3-ium iodide (290mg, 1.00 mmol) and 4-(methylthio)benzaldehyde (140 µL, 1.00 mmol) were dissolved in ethanol (5 mL) inside a round bottom flask. While stirring, one drop of piperidine was added and the resulting solution was refluxed for 12 hours. The reaction was cooled, then diluted with water (10 mL). The resulting precipitate was filtered, rinsed with water and cold ethanol, then dried under vacuum to yield (E)-3-methyl-2-(4-(methylthio)styryl)benzo[d]thiazol-3-ium iodide (341 mg, 80% yield) as a dark orange powder. ^{1}H NMR (400 MHz, DMSO-d_6) δ 8.42 (d, J = 15.6 Hz, 1H), 8.22 (m, 2H), 8.01 (m, 3H), 7.88 (td, J = 7.2, 1.3 Hz, 1H), 7.79 (td, J = 7.2, 1.0 Hz, 1H), 7.43 (d, J = 8.3 Hz, 2H), 4.35 (s, 3H), 2.58 (s, 3H). ^{13}C NMR (100 MHz, DMSO-d_6) δ 172.01, 148.25, 145.12, 142.11, 130.34, 130.25, 129.43, 128.42, 127.83, 125.53, 124.28, 116.87, 112.69, 36.40, 14.05. ESI-MS: *m/z* $C_{17}H_{16}NS_2^+$ calculated: 299.3254, found: $(M)^+$ 299.2941. UV/Vis: Exc. λ_{max} = 415 nm, Em. λ_{max} = 560 nm.

1.2 Fluorescence Measurements

Note that all the concentrations mentioned below represent the final concentrations. The dye, host, and peptide were usually prepared at a minimum of 10× their final concentration, then added into the buffer, salt solution or H_2O, accounting for dilution effects. The mixture was incubated for ~ 30 min at room temperature, then added in the 96-well plate (Product Number 82.1581.120) with a volume = 100 µL. The fluorescence signal (F) was recorded with a BioTek Synergy H1 Hybrid Multi-Mode Microplate Reader at Fluorescence Endpoint read mode with the Ex/Em wavelengths

at 480/600 nm (**4-DSMI**), 540/600 nm (**DTMI**), 480/580 nm (**2-DSMI**), 530/640 nm (**DQMI**), 420/560 nm (**SMITH**), and 435/510 nm (**ThT**), with Gain value = 100.

1) Peptide Screening. The fluorescence (F) emission plots were obtained by using 0.5 μM dye and 4 μM peptide or no peptide in pH 7.4 buffer (20 mM Tris-HCl), or pH 5 buffer (20 mM NaOAc, i.e., CH_3COONa-CH_3COOH). The dye and peptide solutions were prepared at 10× final concentration, and sequentially added into the buffer. F/F_0 values were calculated using F divided by the response of dye in the absence of peptide — F_0 which is defined as the fluorescence recorded for that concentration of dye when [peptide] = 0 μM.

2) Concentration Dependence. The fluorescence titration curves were collected by using 0.5 μM dye and 0 – 10 or 0 – 4 μM alphaB-L peptide in neutral water. The 5 μM dye and 20 μM peptide solutions were sequentially added into water to achieve their final concentrations. The binding affinities were achieved by fitting fluorescence signal by Hill 1 function in Growth/Sigmoidal category from Origin 2021 software.

Hill 1 function is a more general form of Hill function. The equation is:

$$y = START + (END - START)\frac{x^n}{k^n + x^n}$$

x is the ligand concentration, *k* is half-maximal concentration constant, *n* is Hill coefficient.

3) pH Dependence. The fluorescence response plots and curves were gained using 0.5 μM dye with 2 or 4 μM alphaB-L peptide in pH-adjusted H_2O. The fluorescence signals of dye itself without alphaB-L were also measured as comparisons. The pH of water was adjusted in large volume of water with minimal amount of 2 M HCl or NaOH, no buffer was used to avoid additional salts. The dye solution was prepared at 100× final concentration, alphaB-L solution was prepared at its stock or 100× final concentration, then sequentially added into the pH-adjusted H_2O, to minimum the influence on pH.

4) Salt Effects. The salt effects on dye with or without alphaB-L were observed by measuring the fluorescence responses of 0.5 μM dye with 0/4/20/100 μM alphaB-L in 0.5 M salt. The pH values of salt solutions were adjusted to neutral with minimal amount of 2 M HCl or NaOH, except CH_3COONa solution, for which CH_3COOH: H_2O = 1:2 (v/v) solution was used. The dye and

alphaB-L solutions were prepared at a minimum of 30× final concentration, and sequentially added into the salt solution.

5) The 16-Element Array in NaOAc buffer, pH 5. The fluorescence assay was carried out by making the solution containing 0.5 µM fluorescent dye: **DTMI/DQMI/4-DSMI/2-DSMI**, 1 µM cavitand: **TCC/AMI/AMD** or no cavitand, with 4 µM alphaB isomer in 20 mM CH_3COONa-CH_3COOH buffer at pH 5. The dye, host and peptide solutions were prepared at 10× final concentration, and sequentially added into the buffer. F/F_0 values were calculated using F divided by the response of element in the absence of peptide — F_0 which is defined as the fluorescence recorded for **Cav•Dye** when [peptide] = 0 µM.

6) Host•Dye Sensors at pH 7.4. The fluorescence assay was carried out by making the solution containing 0.5 µM fluorescent dye: **DTMI/4-DSMI/2-DSMI**, 2.5 µM cavitand: **TCC** or **AMD**, with 4 µM alphaB isomer in 20 mM Tris-HCl buffer at pH 7.4. The dye, host and peptide solutions were prepared at 10× final concentration. First, the dye and host solutions were premixed and then added to the buffer, after which the peptide solution was introduced. F/F_0 values were calculated using F divided by the response of element in the absence of peptide — F_0 which is defined as the fluorescence recorded for **Cav•Dye** when [peptide] = 0 µM.

7) 12-Element Array with Added Salt. The array was carried out by measuring the fluorescence of the solution containing 0.5 µM fluorescent dye: **DTMI**/**DQMI**/**4-DSMI**, 1 µM host **TCC**, or 0.5 M salt **$NaClO_4$**/**CH_3COONa**, or None, with 2 µM alphaB isomer at each dye's highest response pH (**DTMI**: 7.4, **DQMI**: 6.0, or **4-DSMI**: 8.3) in H_2O. For None or **TCC** elements, the pH of H_2O with minimal amount of 2 M HCl or 2 M NaOH solutions, then **TCC** solution was prepared use this pH-adjusted H_2O. The pH of salt **$NaClO_4$** solution was adjusted with 0.5 M $HClO_4$ or 2 M NaOH solution. The pH of salt **CH_3COONa** solution was adjusted with acetic acid CH_3COOH: H_2O = 1:2 (v/v) solution. The dye and peptide solutions were prepared at 100× final concentration. The dye solution was first premixed with the **TCC** or salt solution, after which the alphaB isomer solution was added. F/F_0 was calculated using the fluorescence normalized against that of blank (F_0 being the fluorescence of element in the absence of peptide).

1.3 Molecular Dynamics Simulations and Interaction Energy Calculations

The Amber20 molecular dynamics package with GPU acceleration was used to conduct MD simulations. The peptide and dye were modeled using ff14sb and GAFF2 force fields,

respectively.[6,7] Ligands 3k are 6MP were parameterized using Amber's antechamber program with the AM1-BCC charge assignment method.[8] The solvated systems were prepared by adding a rectangular box of explicit TIP3P water, extending 12 Å beyond the solute edges, and then adding 3 Na^+ and 2 Cl^- counterions to neutralize the overall system charge. Systems were minimized in four steps. First, using Generalized Born implicit solvent,[9] we minimized the hydrogen atoms, then protein sidechains, and finally the entire protein for 500, 1000, and 5000 steps, respectively. The water was minimized 10000 steps. Next, the entire solvated structure was minimized for 20000 steps. The direct nonbonded energy calculations used a 12 Å cutoff distance, and long-range electrostatics were calculated by the particle mesh Ewald method.[10] The SHAKE algorithm[9] was employed to constrain all bonds involving hydrogen and the simulation timestep was set to 2 fs. Solvated systems were equilibrated in the isothermal-isobaric (NPT) ensemble using the Langevin thermostat. The system was gradually heated from 50 to 275 K in increments of 25 K for 100 ps each, and finally at 298 K for 2000 ps. We carried out three 500ns production run at 298K using different random number seeds, and a frame was saved every 1 ps (i.e. 500,000 frames in total for each raw MD run).

For analysis of the molecular conformations, 3 raw trajectories were resaved every 10 ps using Amber's cpptraj.[11] In addition, we resaved a frame every 500 ps for evaluating intermolecular attractions between ligand and peptide using the molecular mechanics/Poisson-Boltzmann surface area (MM/PBSA) method.[12] This method computes the energy (E) of a system from the peptide, DTMI, and peptide-DTMI complex, and computes the interaction energy as $\Delta < E > = < E_{complex} > - < E_{peptide} > - < E_{DTMI} >$. $< E >$ denotes the computed average energy from a given MD trajectory. The default values of a solute dielectric of 15 and solvent dielectric of 80.0 were used. After careful analysis, the conformation shown in Figures 3d,e displayed the strongest interaction energy ~ -30 kcal/mol and longest association time.

1.4 Data Analysis

The fluorescence emission and F/F_0 bar plots, as well as titration curves were generated with Origin 2021 software. All samples were measured with 3 or 5 repeats, and the average values and standard deviations were reported. Principal Component Analysis (PCA), confidence ellipses, and scatter plots of scaled F/F_0 data were performed with RStudio (Version 1.2.5019), an integrated development environment (IDE) for R (version 3.6.1). Feature selection and classification were

performed with Python 3.9 (64-bit), using StandardScaler for data standardization, Recursive Feature Elimination with Cross-Validation (RFECV) to select the optimal subset of features, Support Vector Machine (SVM) (kernel='linear') as the supervised classification estimator, RFECV(estimator=svm.SVC(kernel='linear'), step=1, cv=StratifiedKFold(n_splits=4, shuffle=True), scoring='accuracy', min_features_to_select=1). Performance metrics for the classification evaluation were calculated by using RepeatedStratifiedKFold (n_splits=4, n_repeats=3) for cross validation. The correlation heatmap of selected features was computed using pandas.DataFrame.corr(method='pearson'). PCA was applied for orthogonal linear transformation and dimensionality reduction, and SVM decision region boundary of PCA plot was generated using plot_decision_regions.

2. NMR Characterization of New Molecules

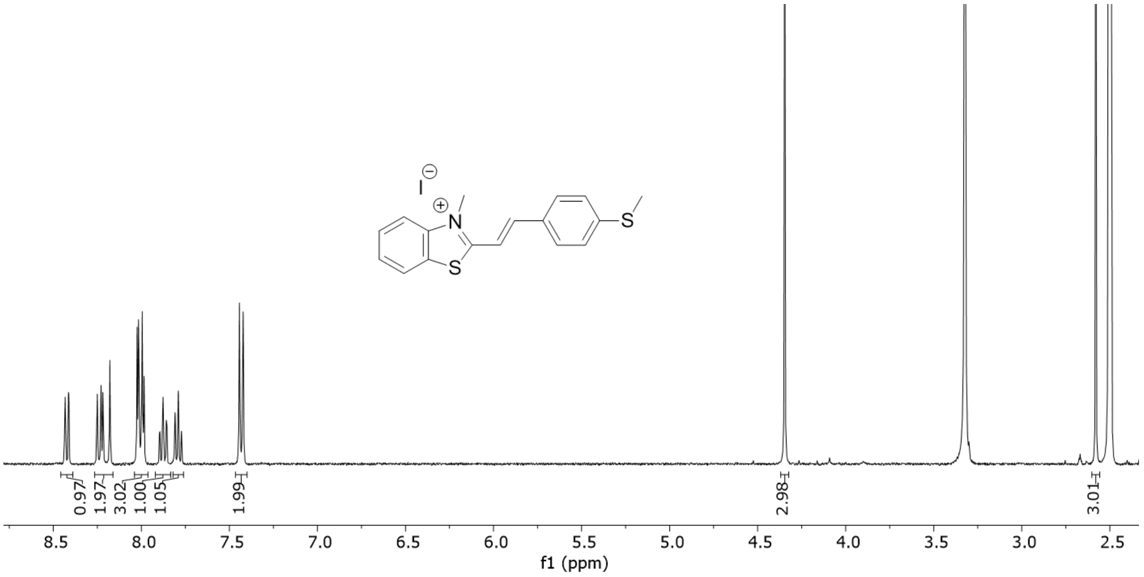

Figure *S*-1. ^{1}H NMR spectrum of **SMITH** (DMSO-d_6, 400 MHz, 298K).

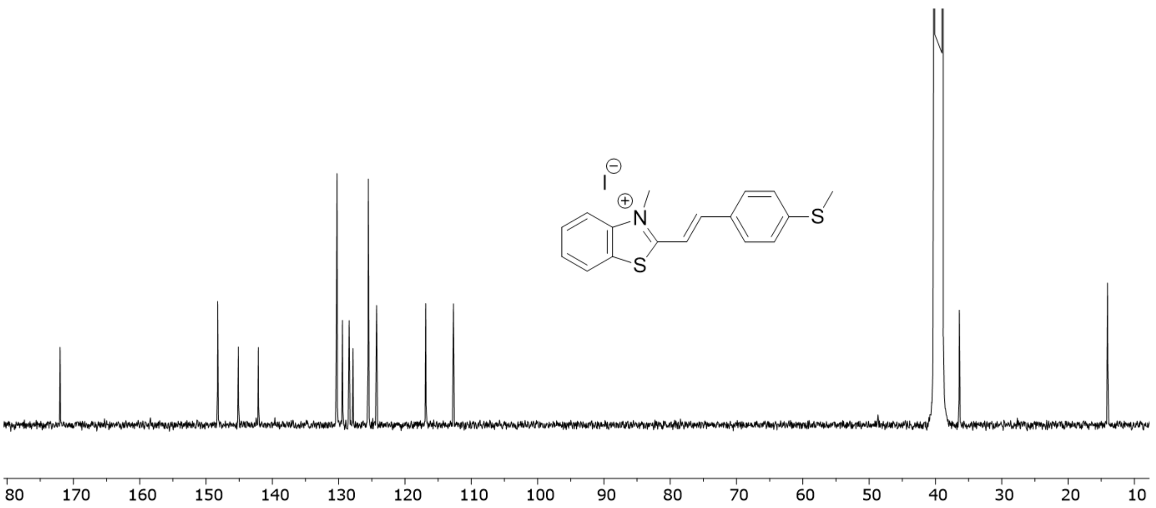

Figure *S*-2. ^{13}C NMR of **SMITH** (DMSO-d_6, 100 MHz, 298K).

3. Peptide Sequences and Asp Isomerization

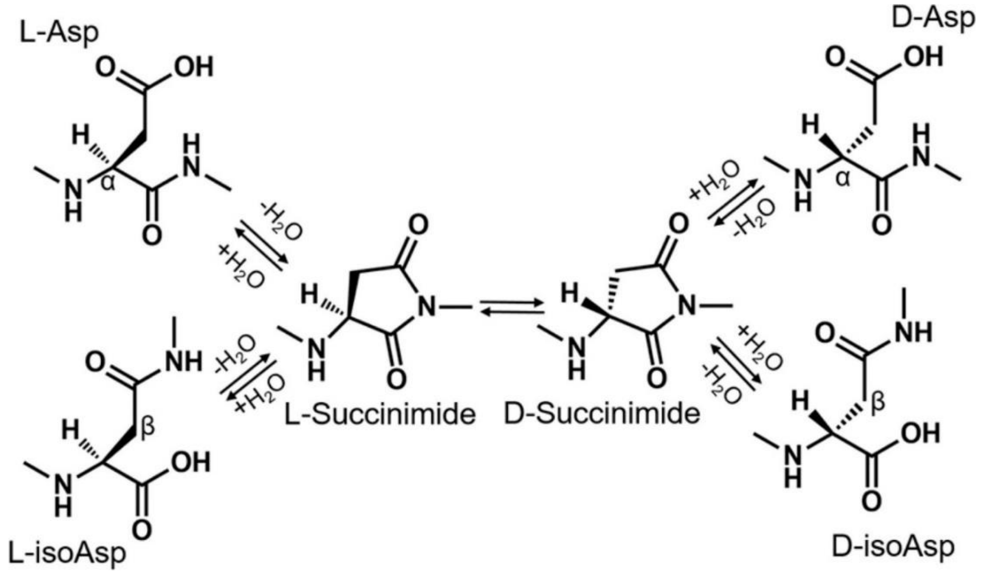

Figure *S*-3. Isomerization mechanism of aspartate residues in long-lived proteins.[13]

Table *S*-1. Peptide sequences used in dye screening experiments. dD indicates the D-Asp epimer modification at the single amino acid position (underlined in sequence).

NO	Name	Sequence	a.a.	pI[a]	Net pH 7.4[a]	GRAVY[a]	Hydrophobicity[b]	M.W.[a]	Net pH 5[a]
1	H3 (1-21)	ARTKQTARKSTGGKAPRKQLA	21	12.71	7	-1.45	2.72	2254.8	7
2	H3 (23-34)	KAARKSAPATGG	12	11.65	3	-0.75	3.56	1114.4	3
3	H3 (73-83)	EIAQDFKTDLR	11	4.31	-1	-0.93	25.19	1335.48	-0.7
4	H3 (1-11)	ARTKQTARKST	11	12.41	4	-1.78	-0.29	1247.42	4
5/6	Tau-L/dD[14, 15]	AKAKT***D***HGAEIVYK	14	9.56	1	-0.73	14.49	1530.72	2.1
7/8	αB-L/dD[16, 17]	APSWF***D***TGLSEMR	13	4.07	-1	-0.49	33.09	1496.63	-0.8
9/10	Aβ-L/dD$_7$ [16]	DAEFRH***D***SGY	10	4.29	-2	-1.61	13.14	1196.18	-0.8

[a] Theoretical isoelectric point (pI), molecular weight (M.W., unit: g/mol), net charge under pH 7.4 or pH 5, and grand average of hydropathicity (GRAVY)[18] of peptides were calculated using Peptide Property Calculator on NovoPro website (https://www.novoprolabs.com/tools/calc_peptide_property).

[b] The hydrophobicity of peptides was obtained using Peptide Synthesis and Proteotypic Peptide Analyzing Tool on Thermo Fisher Scientific website (https://www.thermofisher.com/us/en/home/life-science/protein-biology/peptides-proteins/custom-peptide-synthesis-services/peptide-analyzing-tool.html), which is based on the 'Oleg V. Krokhin' Index and takes into account difficult combinations, such as avoiding too many of the same amino acid in the row and too many hydrophobic amino acids.

Table *S*-2. The alphaB (αB) isomers: all L, D-Asp, L-isoAsp, D-isoAsp, D-Ser, and D-Glu forms.

Source	Sequence	Modification	Ref.
αB-crystallin 57-69 (13 a.a.)	APSWFDTGLSEMR	All L	16, 17, 19, 20
	APSWF***D***TGLSEMR	D-Asp/L-isoAsp/D-isoAsp	
	APSWFDTGL***S***EMR	D-Ser	
	APSWFDTGLS***E***MR	D-Glu	

4. Molecular Dynamics Simulations

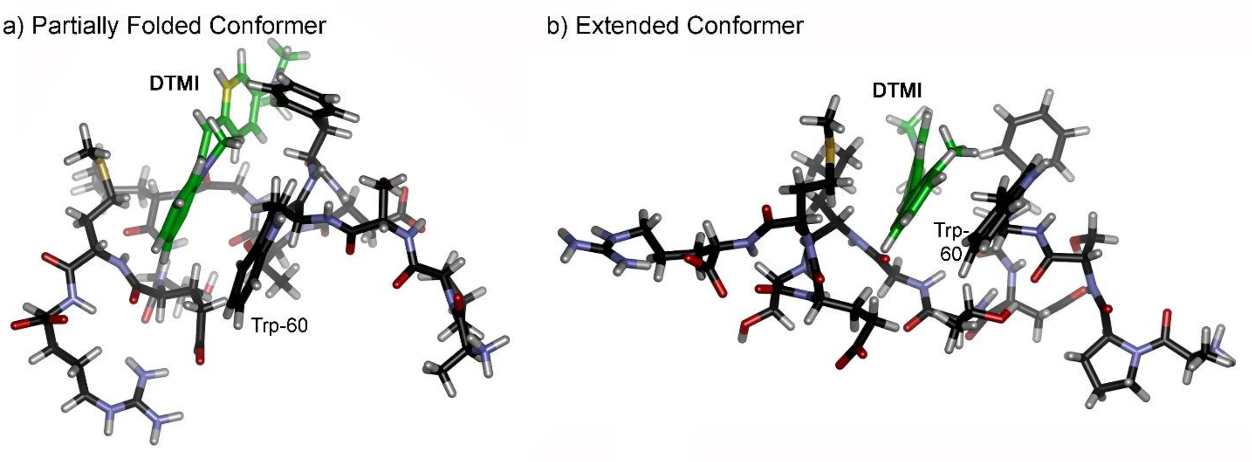

Figure *S*-4. The partially folded conformer and the extended conformer which are calculated to be less stable than the fully folded conformer shown in Figure 3d, e.

5. Dye-Peptide Interaction

5.1 Impacts on Dye (DTMI, DQMI, 4-DSMI, 2-DSMI and SMITH) Fluorescence by Various Peptides in pH 7.4/5 Buffers

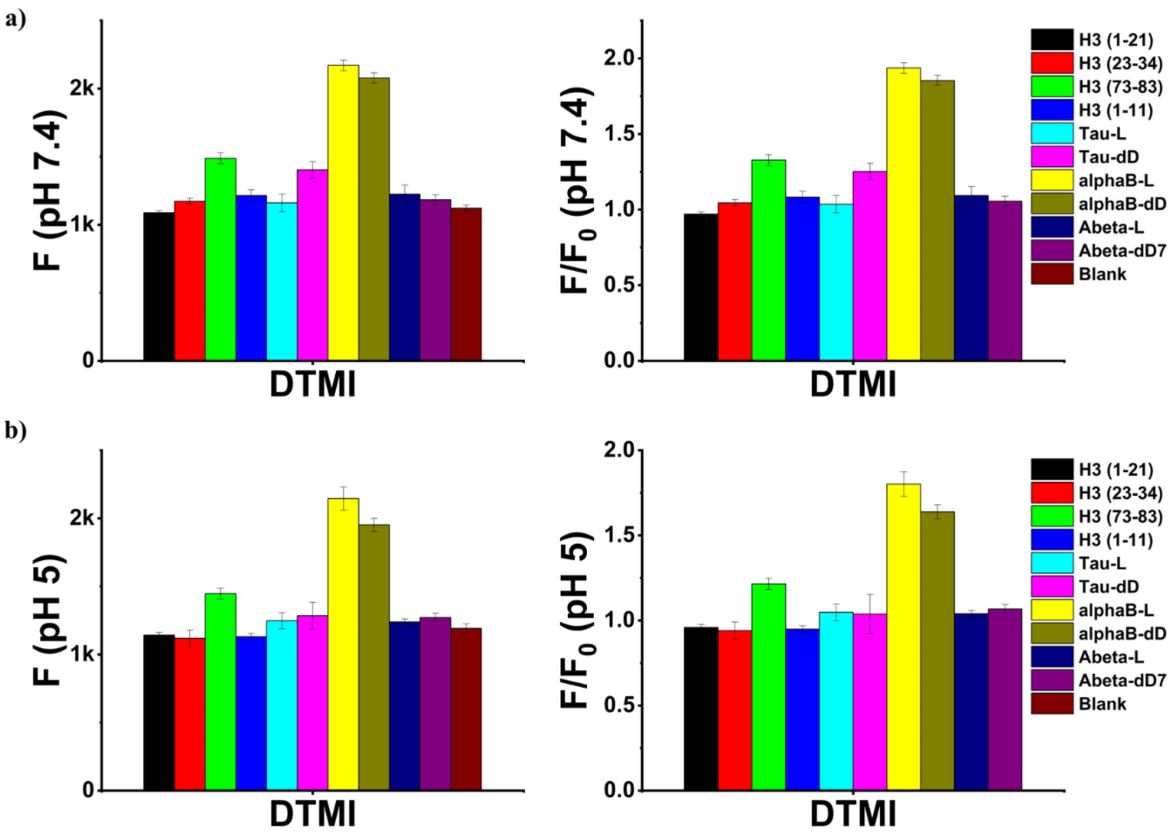

Figure *S*-5. Fluorescence emission and F/F_0 bar plots of peptide screening using **DTMI** in pH 7.4 or pH 5 buffer. [**DTMI**] = 0.5 μM, [peptide] = 4 μM, in 20 mM Tris-HCl buffer, pH 7.4, or 20 mM CH_3COONa-CH_3COOH buffer, pH 5. F/F_0 was calculated using the fluorescence normalized against that of blank (F_0 being the fluorescence of **DTMI** in the absence of peptide).

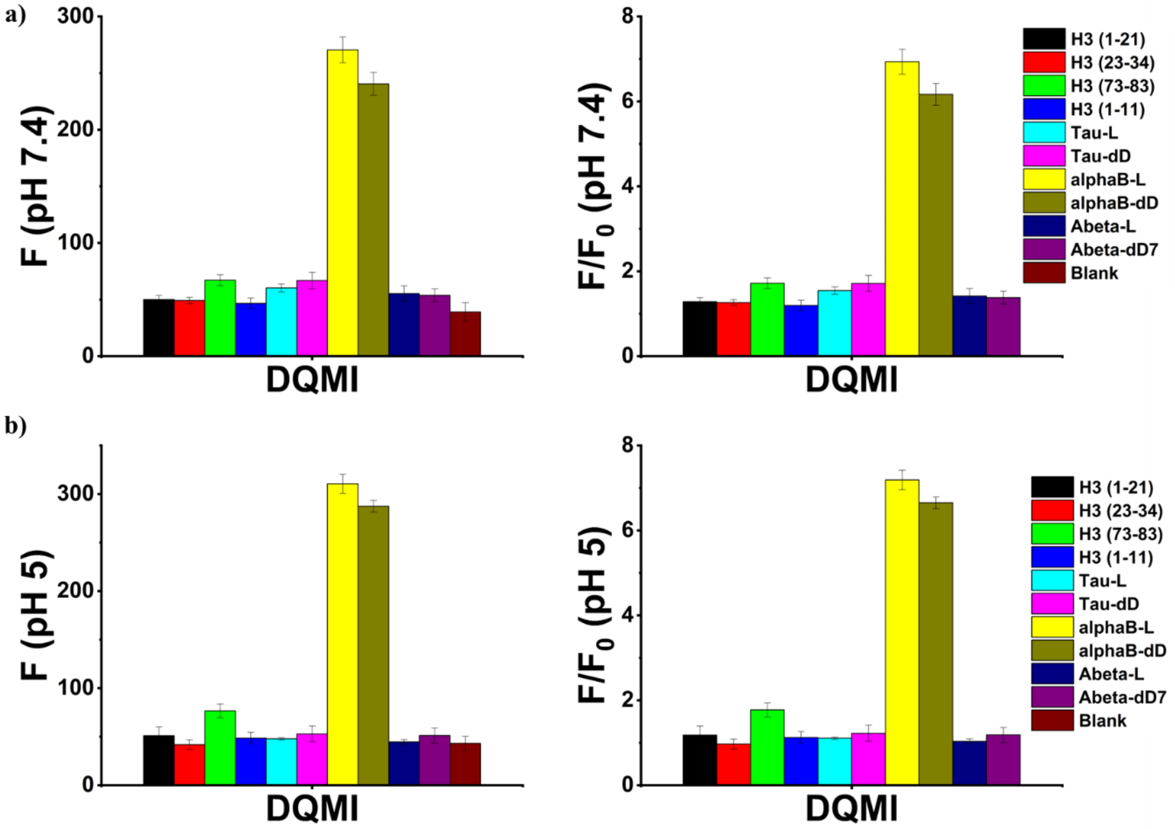

Figure *S*-6. Fluorescence emission and F/F_0 bar plots of peptide screening using **DQMI** in pH 7.4 or pH 5 buffer. [**DQMI**] = 0.5 μM, [peptide] = 4 μM, in 20 mM Tris-HCl buffer, pH 7.4, or 20 mM CH_3COONa-CH_3COOH buffer, pH 5. F/F_0 was calculated using the fluorescence normalized against that of blank (F_0 being the fluorescence of **DQMI** in the absence of peptide).

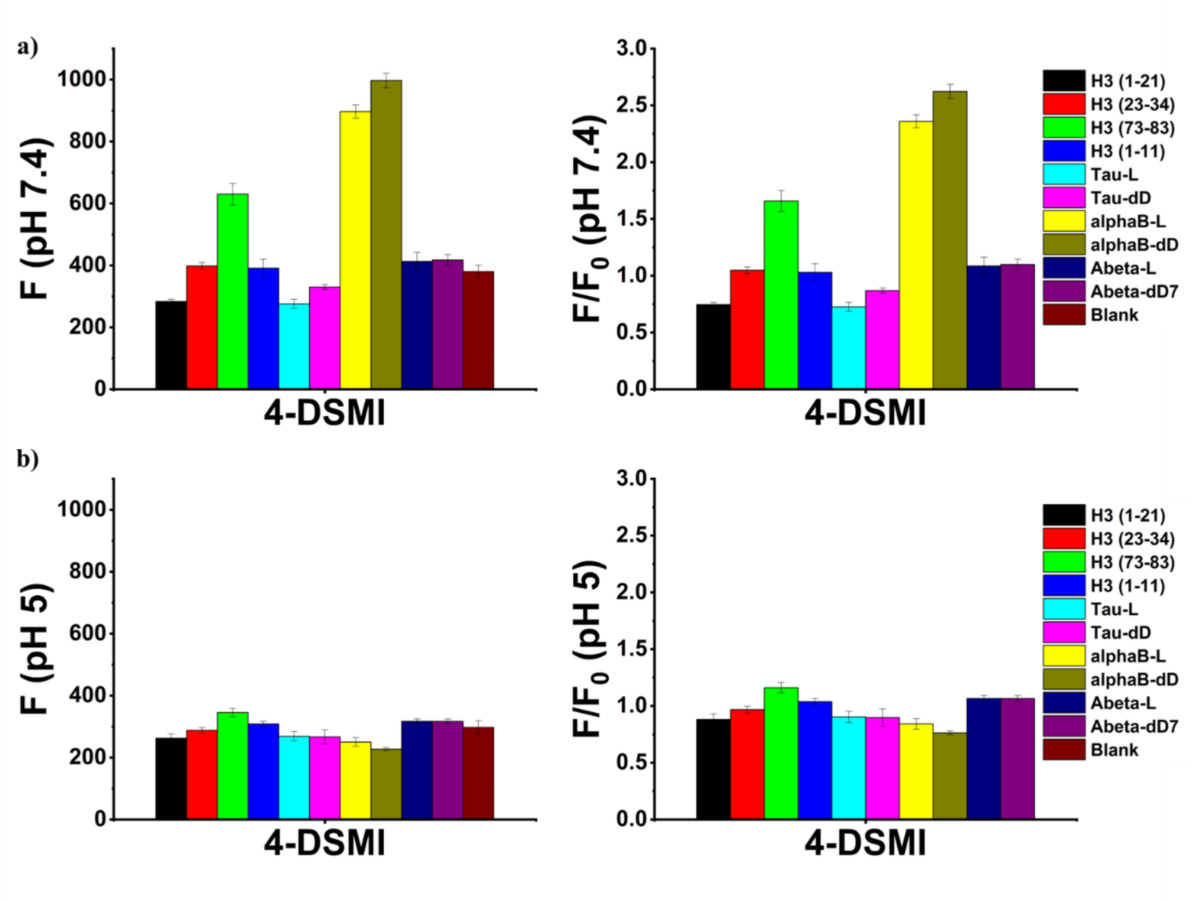

Figure *S*-7. Fluorescence emission and F/F_0 bar plots of peptide screening using **4-DSMI** in pH 7.4 or pH 5 buffer. [**4-DSMI**] = 0.5 μM, [peptide] = 4 μM, in 20 mM Tris-HCl buffer, pH 7.4, or 20 mM CH_3COONa-CH_3COOH buffer, pH 5. F/F_0 was calculated using the fluorescence normalized against that of blank (F_0 being the fluorescence of **4-DSMI** in the absence of peptide).

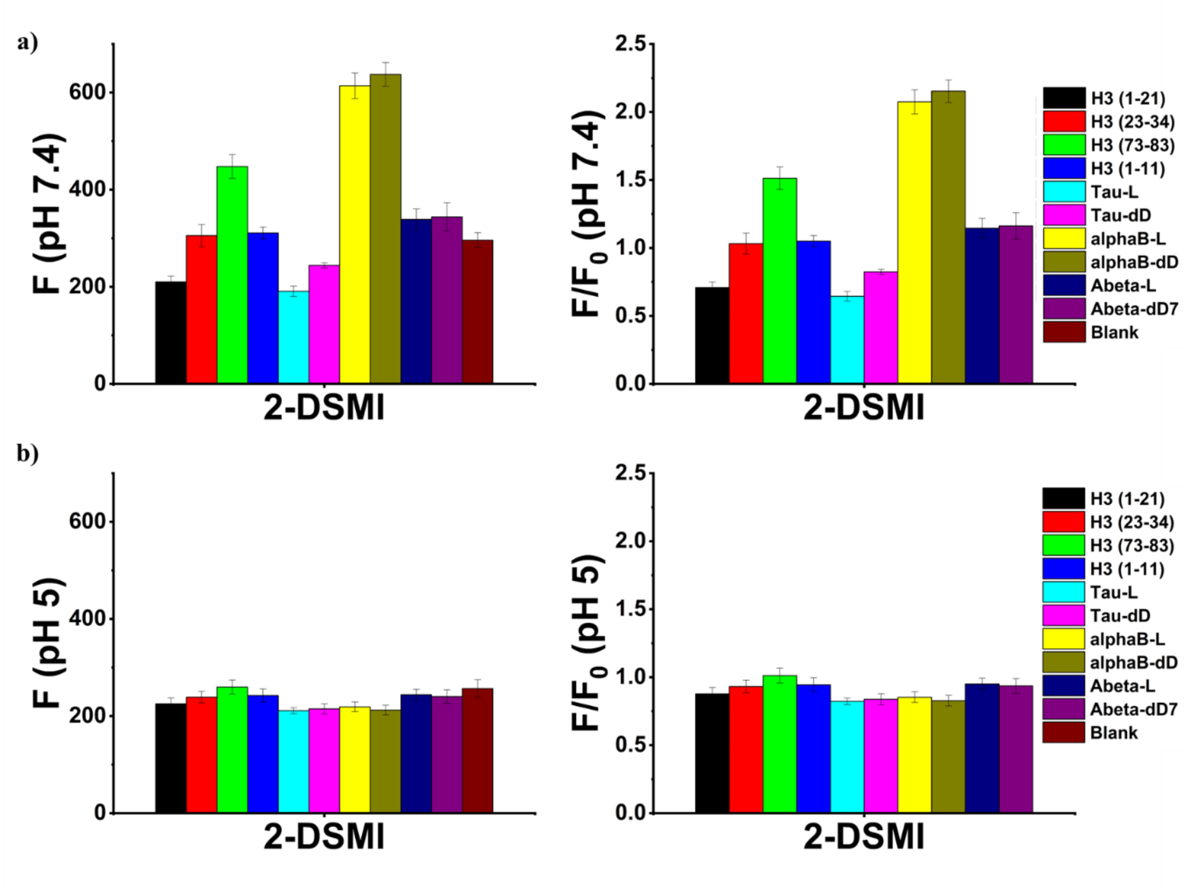

Figure *S*-8. Fluorescence emission and F/F_0 bar plots of peptide screening using **2-DSMI** in pH 7.4 or pH 5 buffer. [**2-DSMI**] = 0.5 μM, [peptide] = 4 μM, in 20 mM Tris-HCl buffer, pH 7.4, or 20 mM CH_3COONa-CH_3COOH buffer, pH 5. F/F_0 was calculated using the fluorescence normalized against that of blank (F_0 being the fluorescence of **2-DSMI** in the absence of peptide).

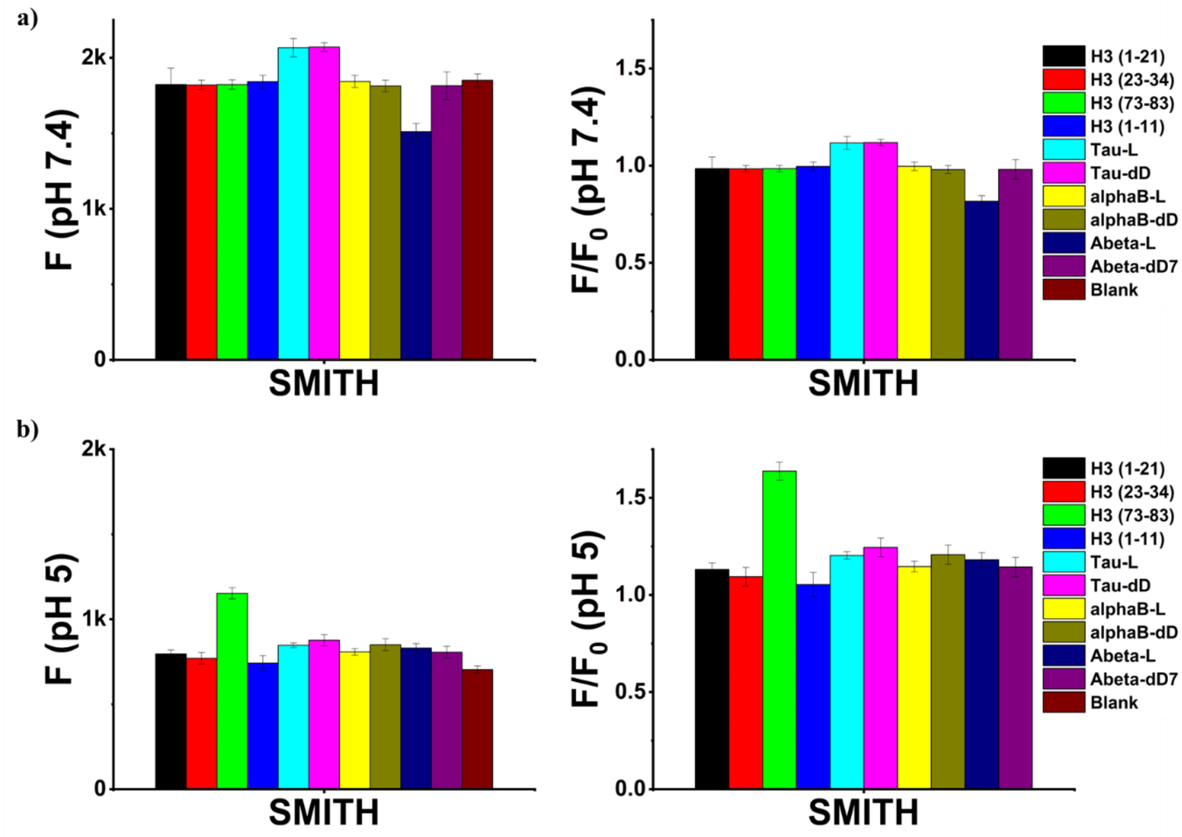

Figure *S*-9. Fluorescence emission and F/F_0 bar plots of peptide screening using **SMITH** in pH 7.4 or pH 5 buffer. [**SMITH**] = 0.5 μM, [peptide] = 4 μM, in 20 mM Tris-HCl buffer, pH 7.4, or 20 mM CH_3COONa-CH_3COOH buffer, pH 5. F and F_0 were the fluorescence of **SMITH** with (F) or without the peptide (F_0).

5.2 Impacts on Dye (DTMI, DQMI, 2-DSMI, 4-DSMI, SMITH, ThT) Fluorescence by Peptide Concentration

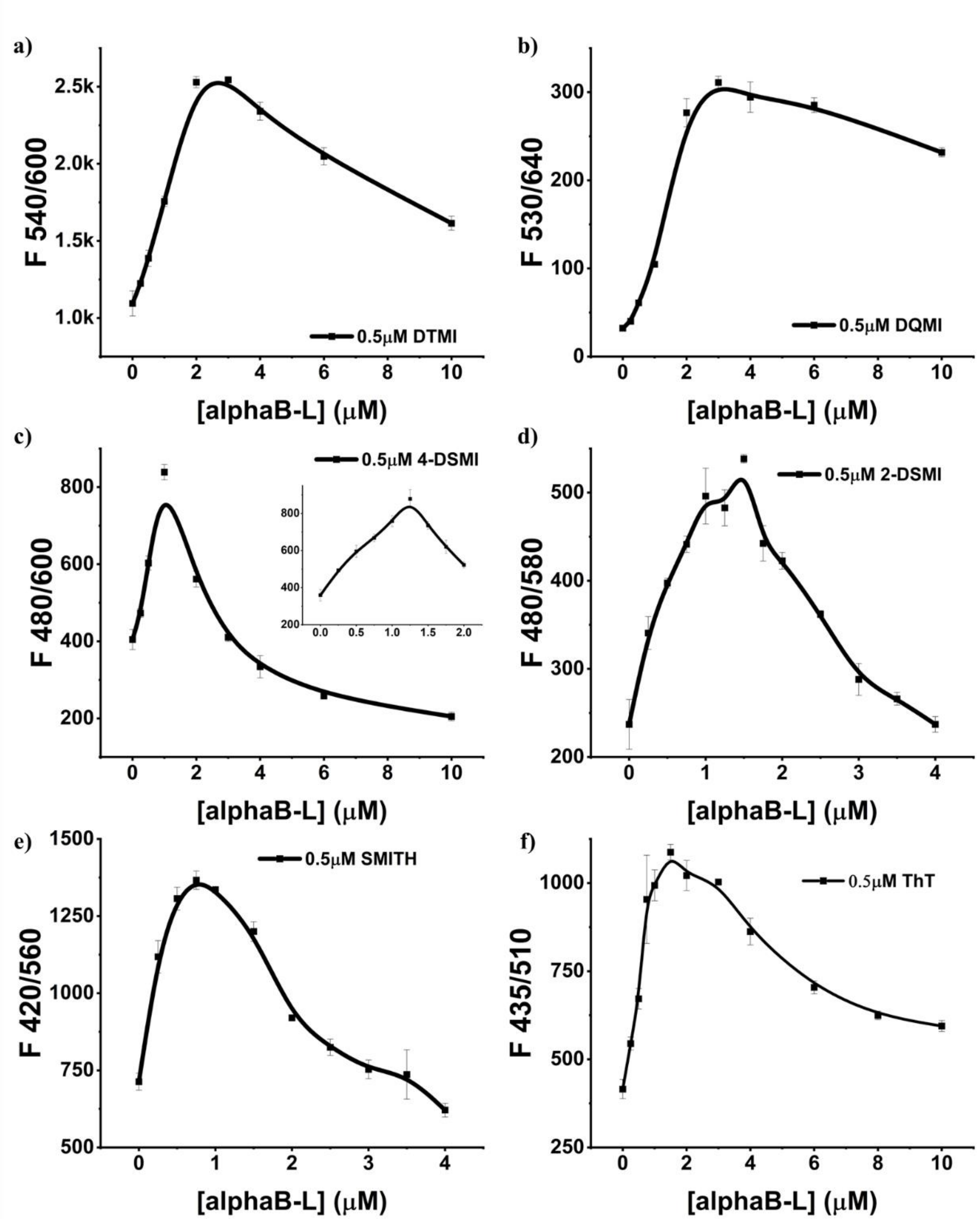

Figure *S*-10. Fluorescence titration curves of 0.5 μM dye: a) **DTMI**, b) **DQMI**, c) **4-DSMI**, d) **2-DSMI**, e) **SMITH**, and f) **ThT** with increasing concentration 0 – 10 or 0 – 4 μM of alphaB-L peptide in neutral H_2O.

Table *S*-3. The Hill 1 fitting result of dye:alphaB-L titration using the data from Figure *S*-10.

Dye:alphaB-L	Increasing	Decreasing
**DTMI**	0−3 µM<br>k 1.1 ± 0.1<br>n 4.7 ± 2.4	2−10 µM<br>k 5.9 ± 0.8<br>n 4.5 ± 2.8
**DQMI**	0−3µM<br>k 1.9 ± 1.1<br>n 2.4 ± 1.3	NA
**4-DSMI**	0.25−1.5 µM (remove the data point at 1.25 µM)<br>k 0.57 ± 0.11<br>n 3.6 ± 3.1	1−10 µM<br>k 1.5 ± 0.1<br>n 1.7 ± 0.1
**2-DSMI**	0.25−1.25 µM<br>k 0.60 ± 0.15<br>n 2.3 ± 1.7	1.75−4 µM<br>k 2.7 ± 0.1<br>n 4.8 ± 1.6
**SMITH**	0−1 µM<br>k 0.20 ± 5.7<br>n 2.0 ± 26.1	0.75−4 µM<br>k 1.8 ± 0.1<br>n 4.1 ± 1.2

5.3 Effects from pH on Dye (DTMI, DQMI, and 4-DSMI) Fluorescence

5.3.1 Dyes with 4 µM alphaB-L in pH-adjusted H_2O

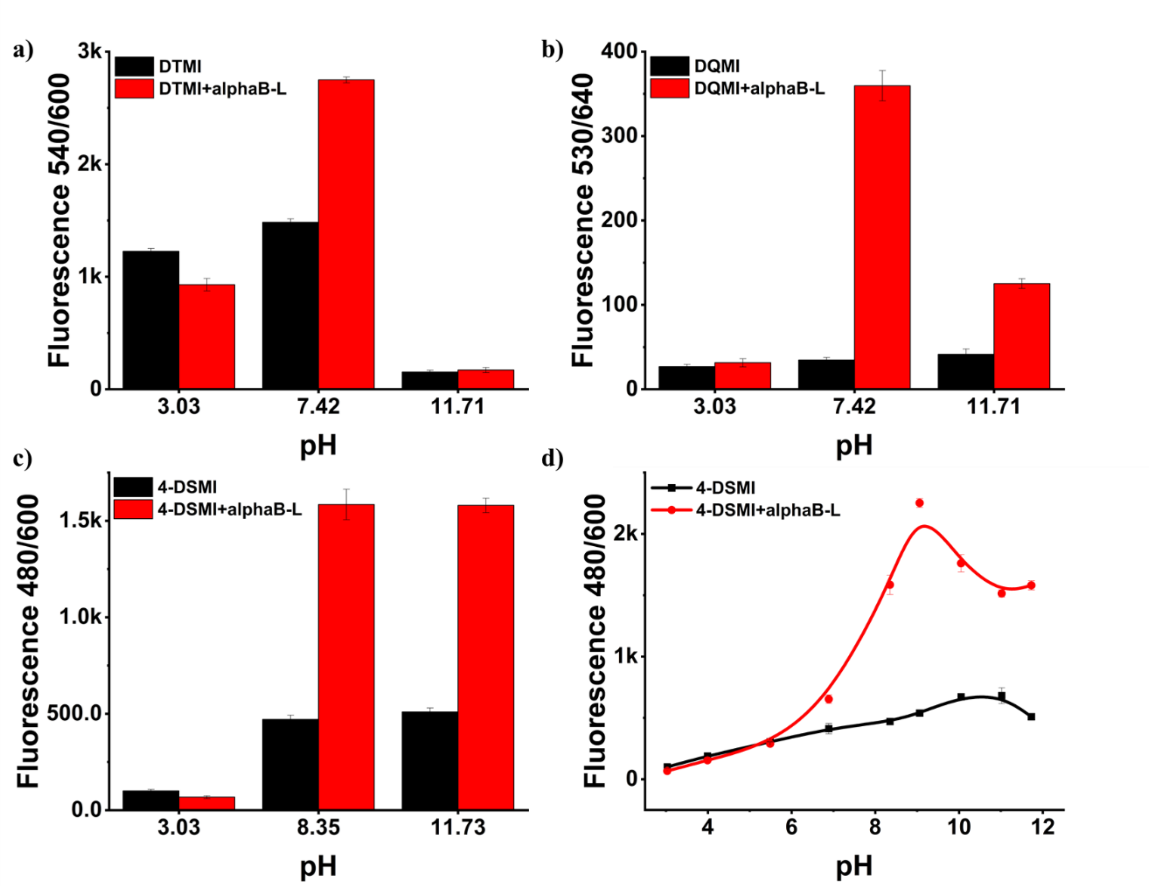

Figure *S*-11. Fluorescence emission plots of 0.5 µM dye: a) **DTMI**, b) **DQMI**, c) and d) **4-DSMI** with or without 4 µM alphaB-L peptide in acidic, neutral, and basic pH water solutions. pH was adjusted in large volume of water with minimal amount of HCl or NaOH, no buffer was used to avoid additional salts.

5.3.2 Dyes with 2 μM alphaB-L in pH-adjusted H_2O

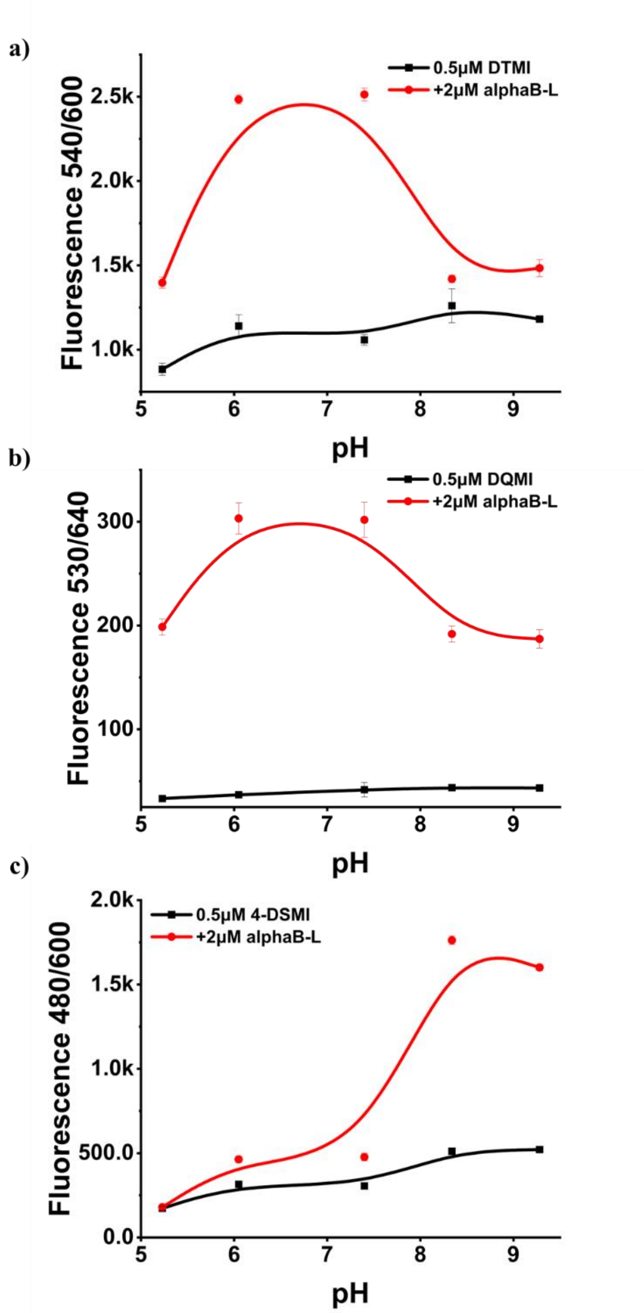

Figure *S*-12. Fluorescence emission response curves of 0.5 μM dye: a) **DTMI**, b) **DQMI**, and c) **4-DSMI** with or without 2 μM alphaB-L peptide in pH-adjusted H_2O. pH was adjusted in large volume of water with minimal amount of 2 M HCl or NaOH, no buffer was used to avoid additional salts.

5.4 Effects from Salts on Dye (DTMI, DQMI, and 4-DSMI) Fluorescence

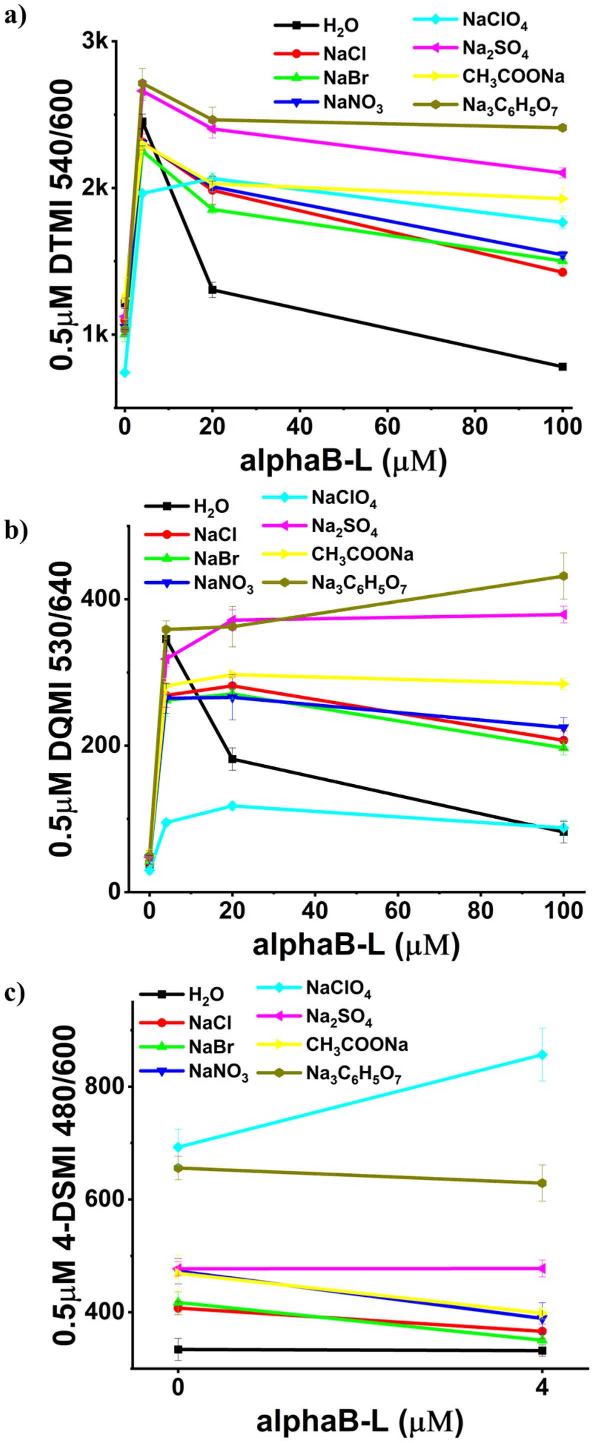

Figure *S*-13. Fluorescence emission titration curves of a) **DTMI**, b) **DQMI**, and c) **4-DSMI** with or without alphaB-L peptide in salt solutions. [Dye] = 0.5 μM, [alphaB-L] = 0/4/20/100 μM, [Salt] = 0.5 M, in H_2O with pH adjusted to neutral.

6. Array Sensing of AlphaB Isomers

6.1 Host•Dye (DTMI/4-DSMI/2-DSMI) Sensors at pH 7.4

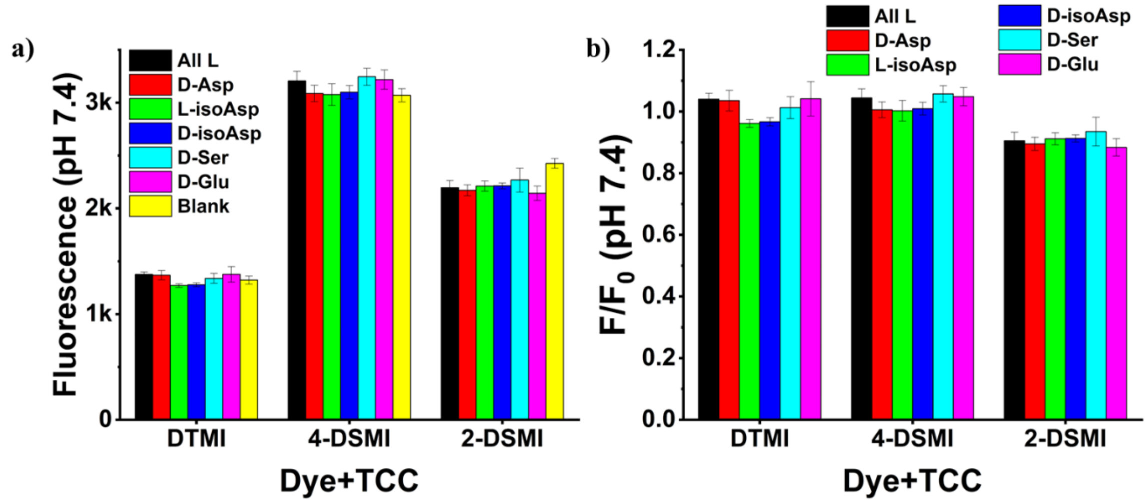

Figure *S*-14. a) Fluorescence emission and b) F/F_0 bar plots of **TCC•Dye** elements: **DTMI**, **4-DSMI**, and **2-DSMI** for sensing alphaB 6 isomers: all L, D-Asp, L-isoAsp, D-isoAsp, D-Ser, and D-Glu forms. [Dye] = 0.5 μM, [**TCC**] = 2.5 μM, [alphaB isomer] = 4 μM, in 20 mM Tris-HCl buffer, pH 7.4. F/F_0 was calculated using the fluorescence normalized against that of blank (F_0 being the fluorescence of **TCC•Dye** in the absence of peptide).

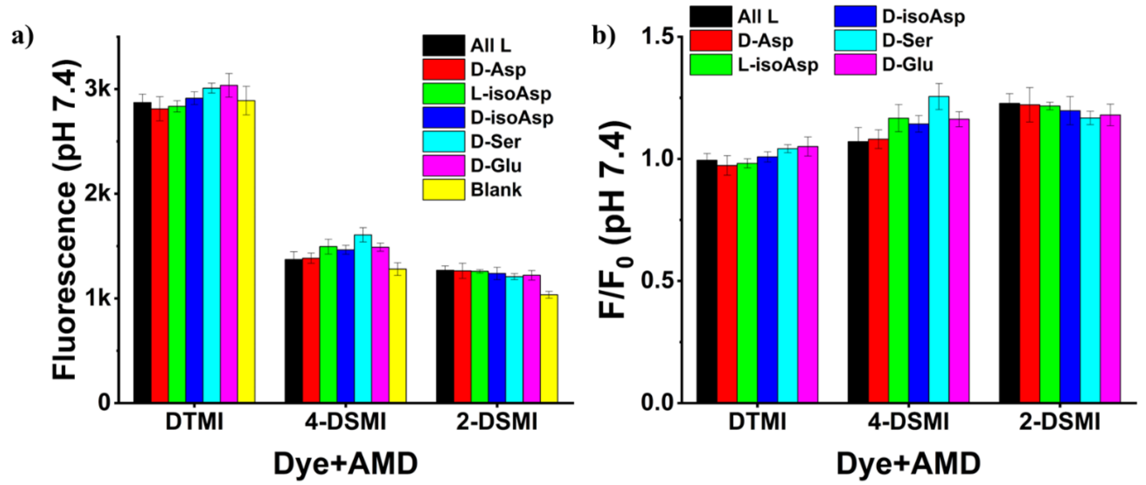

Figure *S*-15. a) Fluorescence emission and b) F/F_0 bar plots of **AMD•Dye** elements: **DTMI**, **4-DSMI**, and **2-DSMI** for sensing alphaB 6 isomers: all L, D-Asp, L-isoAsp, D-isoAsp, D-Ser, and D-Glu forms. [Dye] = 0.5 μM, [**AMD**] = 2.5 μM, [alphaB isomer] = 4 μM, in 20 mM Tris-HCl buffer, pH 7.4. F and F_0 were the fluorescence of **AMD•Dye** with (F) or without the peptide (F_0).

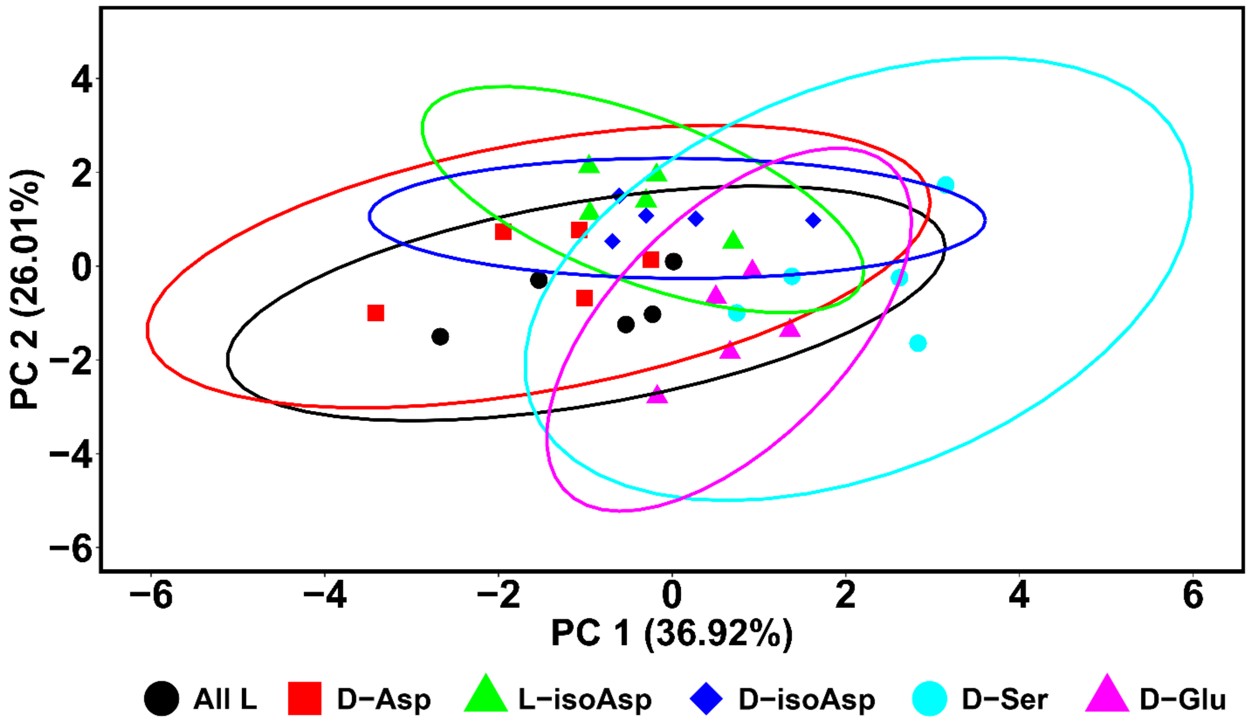

Figure *S*-16. PCA score plot of 6 alphaB isomers, obtained with the data of 6 **Host•Dye** sensors at pH 7.4 in Figures *S*-14 and *S*-15: dye **DTMI/4-DSMI/2-DSMI** with host **TCC**/**AMD**. [Dye] = 0.5 µM, [Host] = 2.5 µM, [alphaB isomer] = 4 µM, buffer 20 mM Tris-HCl at pH 7.4. Ellipses indicate 95% confidence.

6.2 The 12-Element Array with Added Salts

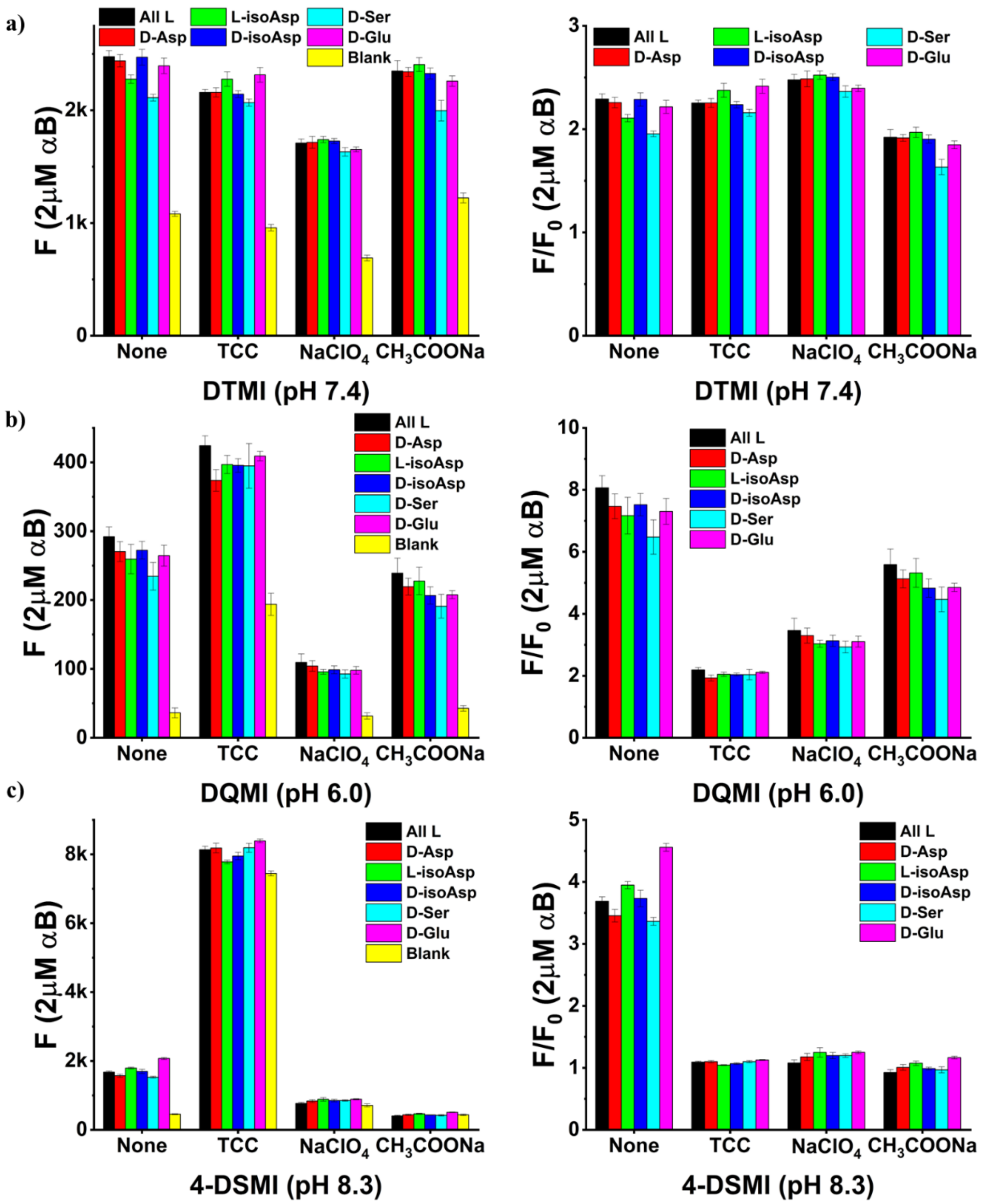

Figure *S*-17. Fluorescence emission (left) and F/F_0 (right) bar plots of 12-element array: a) **DTMI**, b) **DQMI** and c) **4-DSMI** with None/host **TCC**/salt **$NaClO_4$/CH_3COONa** at variable pH (**DTMI**: 7.4, **DQMI**: 6.0, or **4-DSMI**: 8.3) in H_2O for sensing alphaB 6 isomers: all L, D-Asp, L-isoAsp, D-isoAsp, D-Ser, and D-Glu forms. [Dye] = 0.5 μM, [**TCC**] = 1 μM, [**$NaClO_4$**] = [**CH_3COONa**] = 0.5 M, and [alphaB isomer] = 2 μM. F and F_0 were the fluorescence of each sensing element with (F) or without the peptide (F_0).

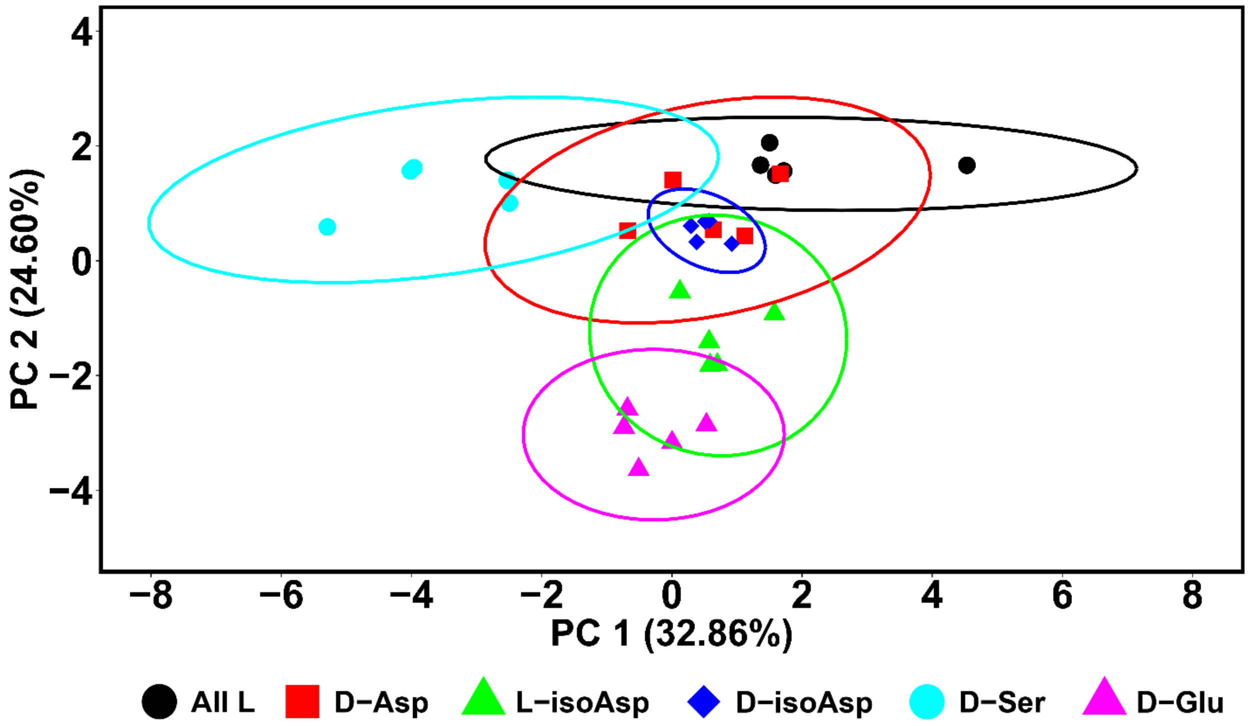

Figure *S*-18. PCA score plot of 6 alphaB isomers, obtained with the data of 12-element array in Figure *S*-17: dye **DTMI/DQMI/4-DSMI** with None/host **TCC**/salt **$NaClO_4$/CH_3COONa**. [Dye] = 0.5 µM, [**TCC**] = 1 µM, [**$NaClO_4$**] = [**CH_3COONa**] = 0.5 M, [alphaB isomer] = 2 µM, pH values of water solutions were adjusted to ~7.4 for **DTMI** elements, ~6.0 for **DQMI** elements, and ~8.3 for **4-DSMI** elements, respectively. Ellipses indicate 95% confidence.

6.3 The 16-Element Array at pH 5

6.3.1 The Fluorescence Profile of All the 16 Elements

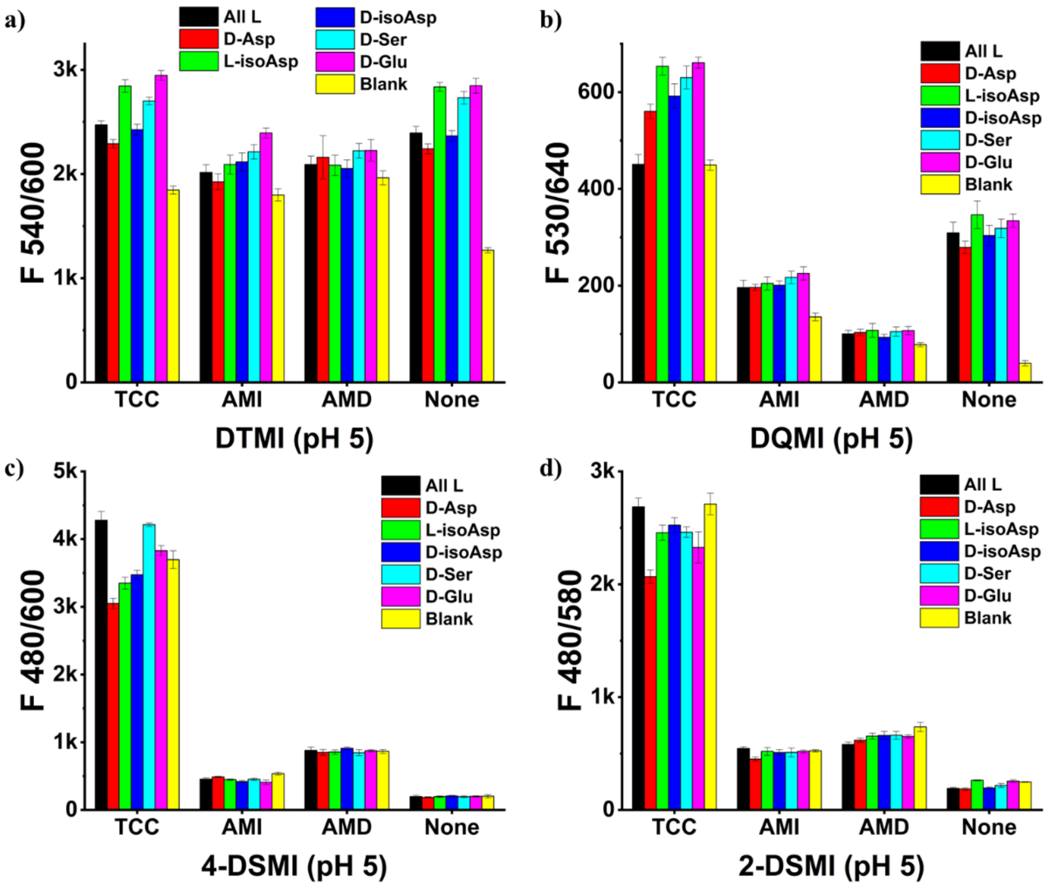

Figure *S*-19. Fluorescence emission bar plots of a 16-element pH 5 array: dye a) **DTMI**, b) **DQMI**, c) **4-DSMI**, and d) **2-DSMI** with host **TCC/AMI/AMD** or no cavitand for sensing 6 alphaB isomers. [Dye] = 0.5 μM, [Host] = 1 μM, [alphaB isomer] = 4 μM, in 20 mM CH_3COONa-CH_3COOH buffer at pH 5.

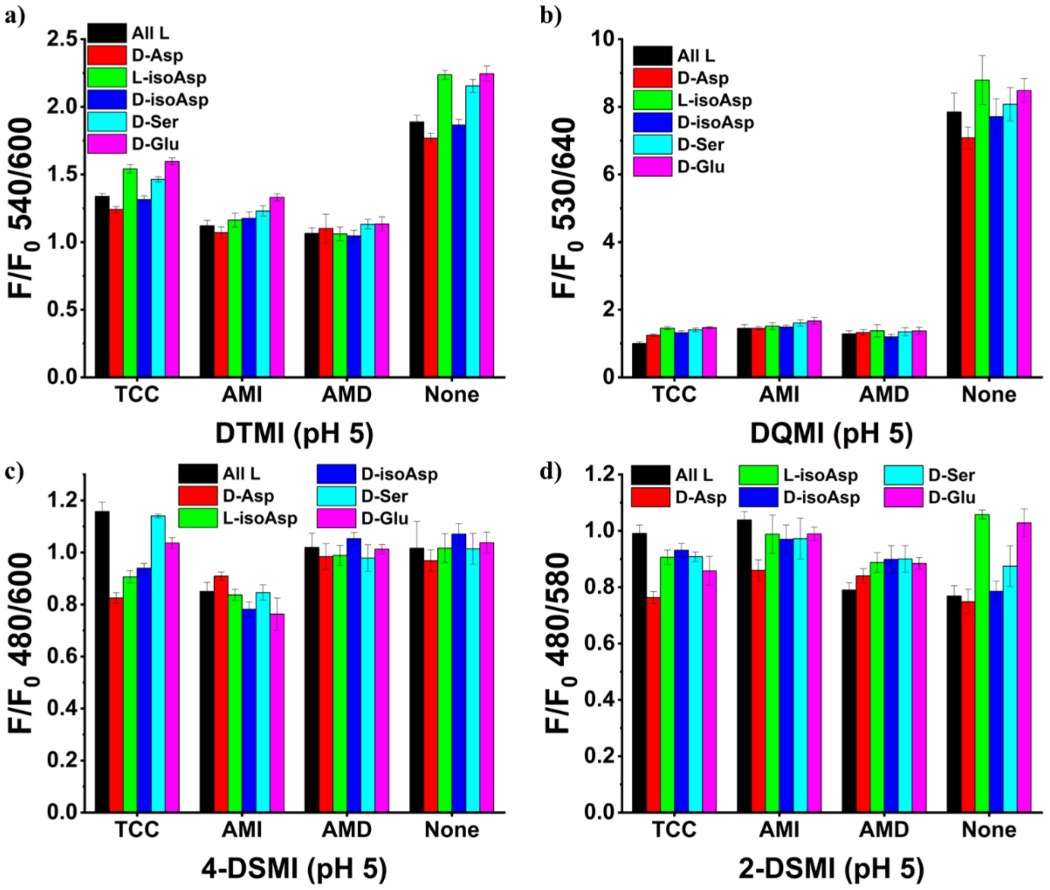

Figure *S*-20. The F/F_0 bar plots of a 16-element pH 5 array: dye a) **DTMI**, b) **DQMI**, c) **4-DSMI**, and d) **2-DSMI** with host **TCC/AMI/AMD/None** for sensing 6 alphaB isomers. [Dye] = 0.5 μM, [Host] = 1 μM, [alphaB isomer] = 4 μM, in 20 mM CH_3COONa-CH_3COOH buffer, pH 5. F and F_0 were the fluorescence of each sensing element with (F) or without the peptide (F_0) as shown in Figure *S*-19.

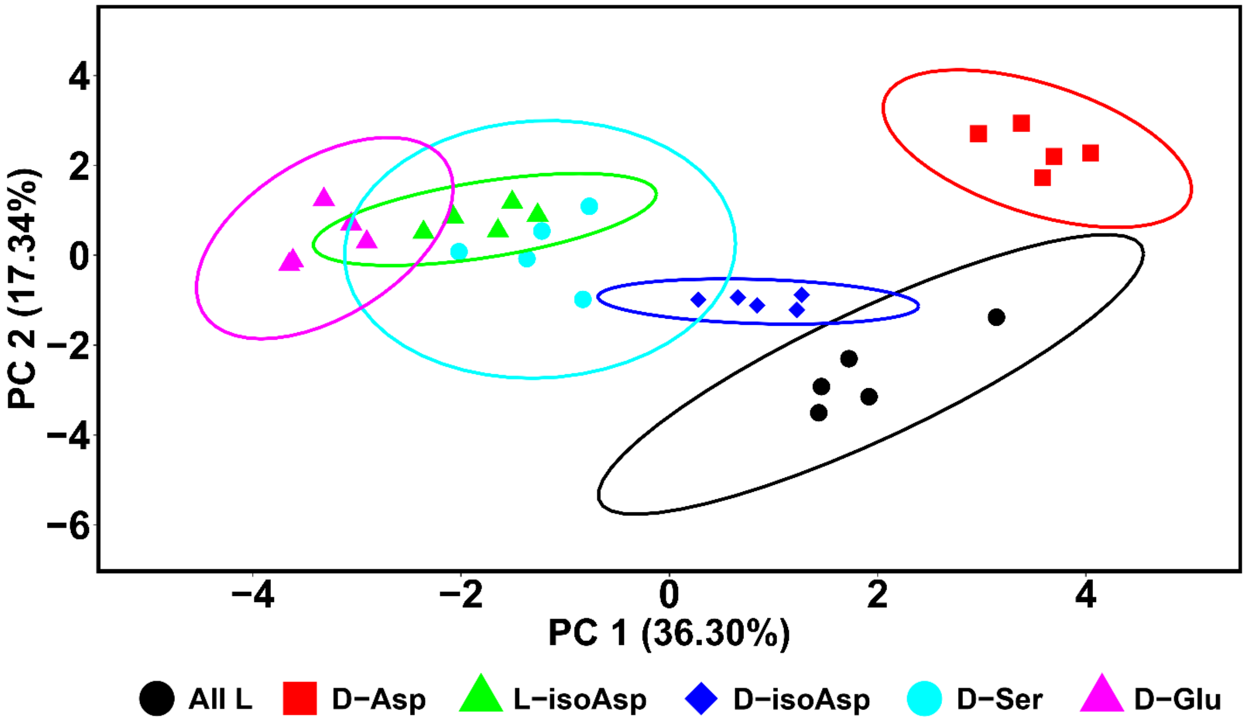

Figure *S*-21. The PCA score plot for isomer discrimination using the data in Figure *S*-20 of 16-element array collected in pH 5 buffer. Ellipses indicate 95% confidence.

6.3.2 SVM-RFECV of Six Potential Elements in pH 5 Buffer

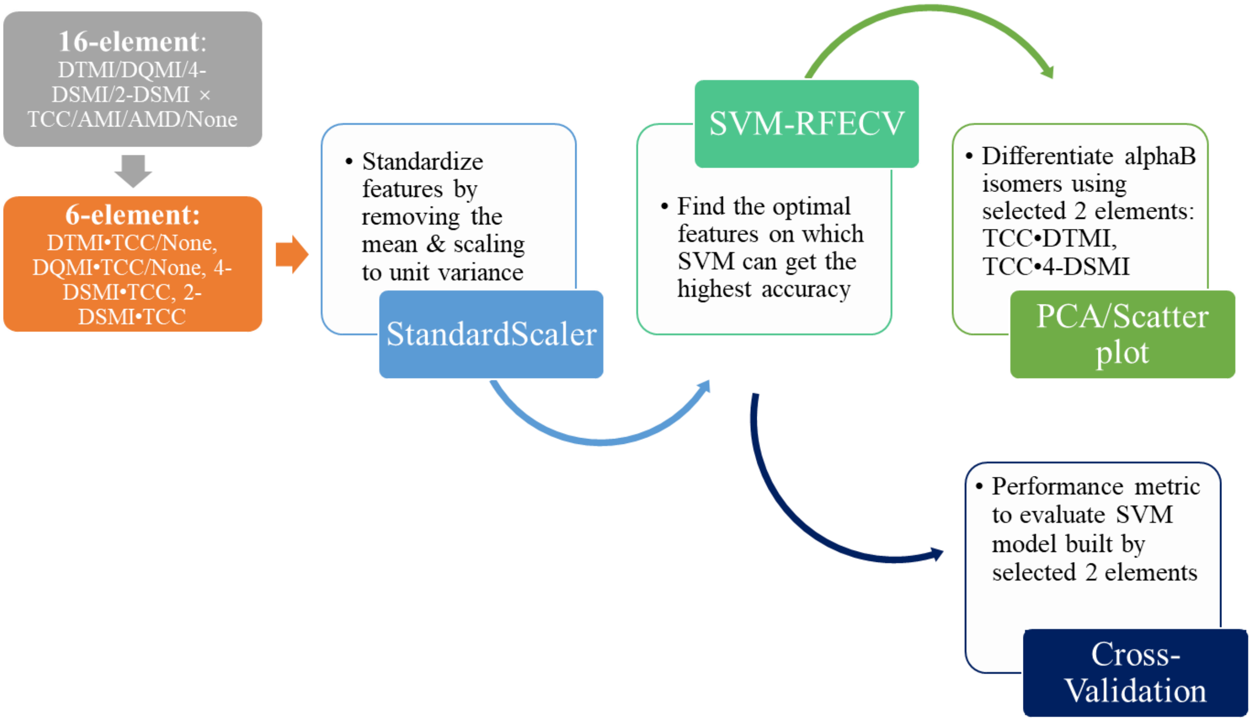

Figure *S*-22. Operational flowchart of the SVM-RFECV machine learning approach for feature selection.

Table *S*-4. SVM-RFECV rank list of 6-element array: **TCC•4-DSMI, TCC•DTMI, DTMI, TCC•2-DSMI, TCC•DQMI,** and **DQMI**, in pH 5 buffer for classification of 6 alphaB isomers.

Element	Rank	Select
**TCC•4-DSMI**	1	True
**TCC•DTMI**	1	True
**DTMI**	2	False
**TCC•2-DSMI**	3	False
**TCC•DQMI**	4	False
**DQMI**	5	False

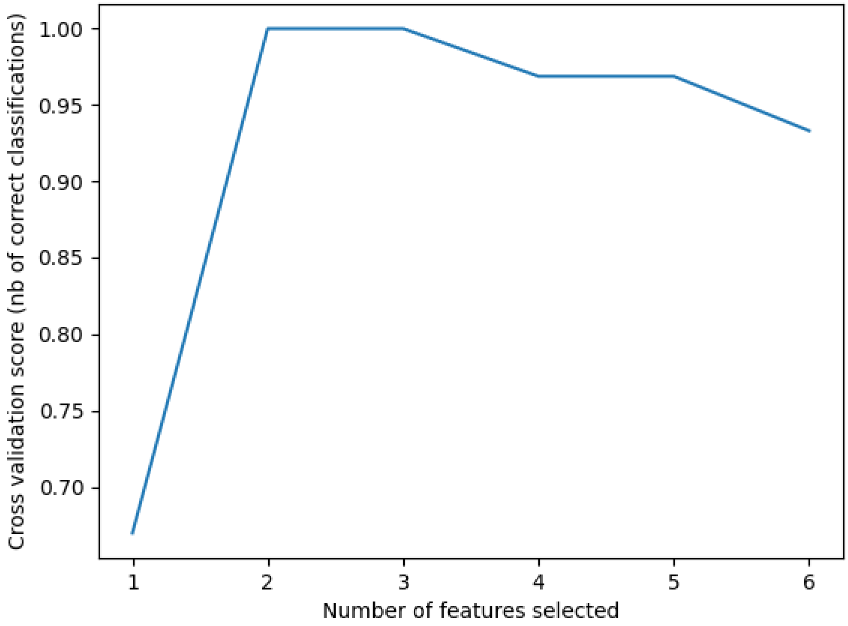

Figure *S*-23. The cross-validation scores correspond to the increasing numbers of features from the 6-element array for 6 alphaB isomers classification.

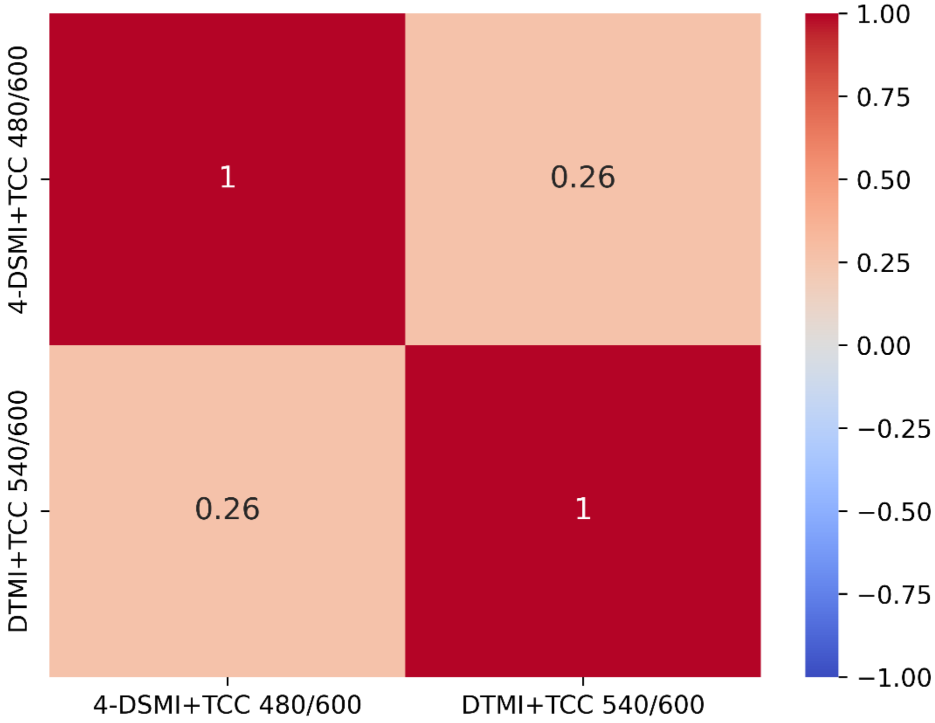

Figure *S*-24. Correlation heatmap of SVM-RFECV selected 2 features: **TCC•4-DSMI** and **TCC•DTMI** in the data set of 6 alphaB isomers in pH 5 buffer.

Table *S*-5. The 3 repeated 4-fold cross-validation scores of SVM-RFECV selected 2 elements **TCC•DTMI** and **TCC•4-DSMI** from 6-element array in pH 5 buffer, with SVM as the estimator for classification of 6 alphaB isomers.

**Evaluation Metrics**	**Score (standard deviation from 3 repeated running of the 4-fold cross validation)**
Accuracy	1.0000 (0.0000)
Sensitivity	1.0000 (0.0000)
Specificity	1.0000 (0.0000)
Precision	1.0000 (0.0000)
F1 Score	1.0000 (0.0000)
AUC	1.0000 (0.0000)

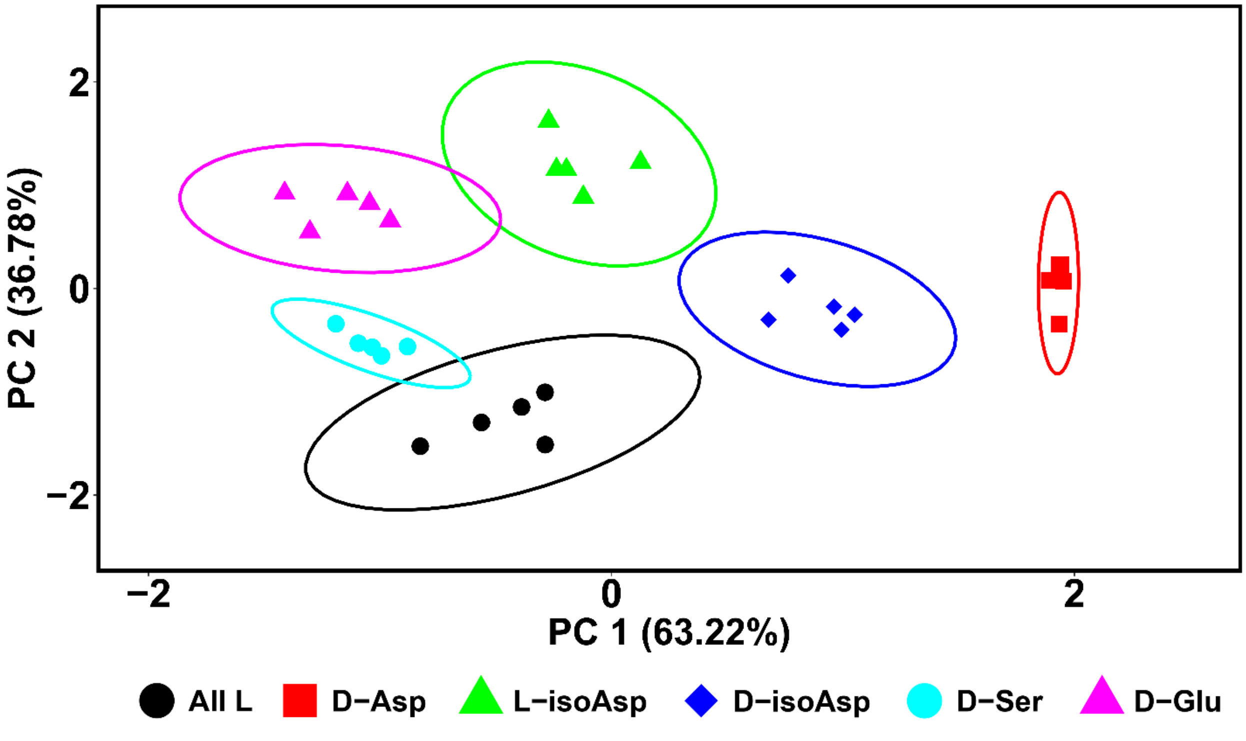

Figure *S*-25. The PCA score plot for isomer discrimination using the 2 most optimal elements selected by SVM-RFECV: **TCC•4-DSMI** and **TCC•DTMI**. Ellipses indicate 95% confidence.

6.3.3 SVM-RFECV of 16-Element pH 5 Array for 4 L/D Isomers

Table *S*-6. SVM-RFECV rank list of 16-element array in pH 5 buffer for classification of 4 alphaB peptides: All L, D-Asp, D-Ser, and D-Glu forms.

Dye	Host	Rank	Select
**4-DSMI**	**TCC**	5	False
	**AMI**	8	False
	**AMD**	12	False
	**None**	15	False
**DTMI**	**TCC**	1	True
	**AMI**	3	False
	**AMD**	16	False
	**None**	6	False
**2-DSMI**	**TCC**	2	False
	**AMI**	11	False
	**AMD**	10	False
	**None**	7	False
**DQMI**	**TCC**	4	False
	**AMI**	9	False
	**AMD**	14	False
	**None**	13	False

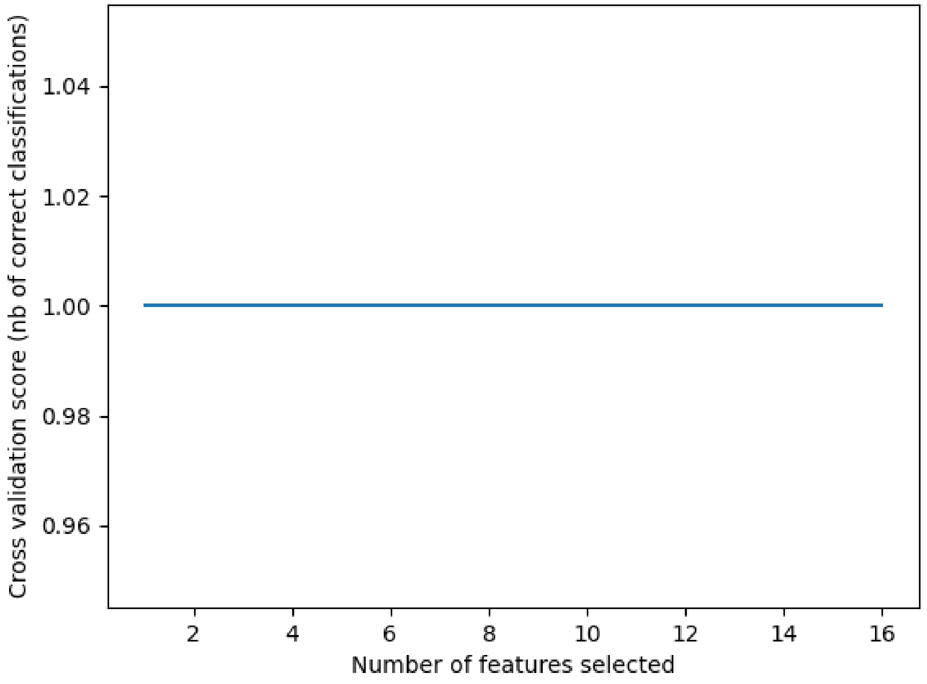

Figure *S*-26. The cross-validation scores correspond to the increasing numbers of features from the 16-element array for 4 alphaB L/D isomers classification.

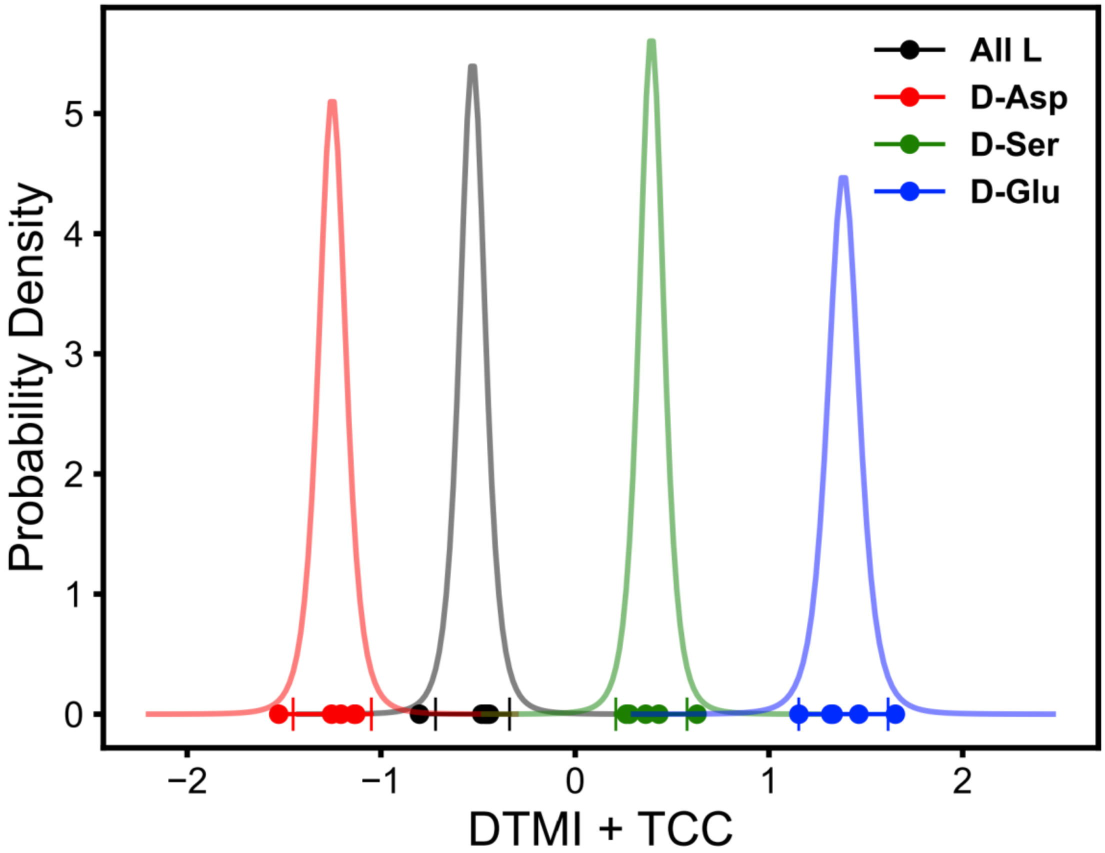

Figure *S*-27. *t*-distribution plot of 4 alphaB L/D isomers using the SVM-RFECV selected feature: **TCC•DTMI** from the 16-element array in pH 5 buffer, vertical markers = 95% confidence intervals.

Table *S*-7. The 3 repeated 4-fold cross-validation scores of SVM-RFECV selected feature **TCC•DTMI** from 16-element array in pH 5 buffer, with SVM as the estimator for classification of 4 alphaB L/D isomers: All L, D-Asp, D-Ser, and D-Glu forms.

Evaluation Metrics	Score (standard deviation from 3 repeated running of the 4-fold cross validation)
Accuracy	1.0000 (0.0000)
Sensitivity	1.0000 (0.0000)
Specificity	1.0000 (0.0000)
Precision	1.0000 (0.0000)
F1 Score	1.0000 (0.0000)
AUC	1.0000 (0.0000)

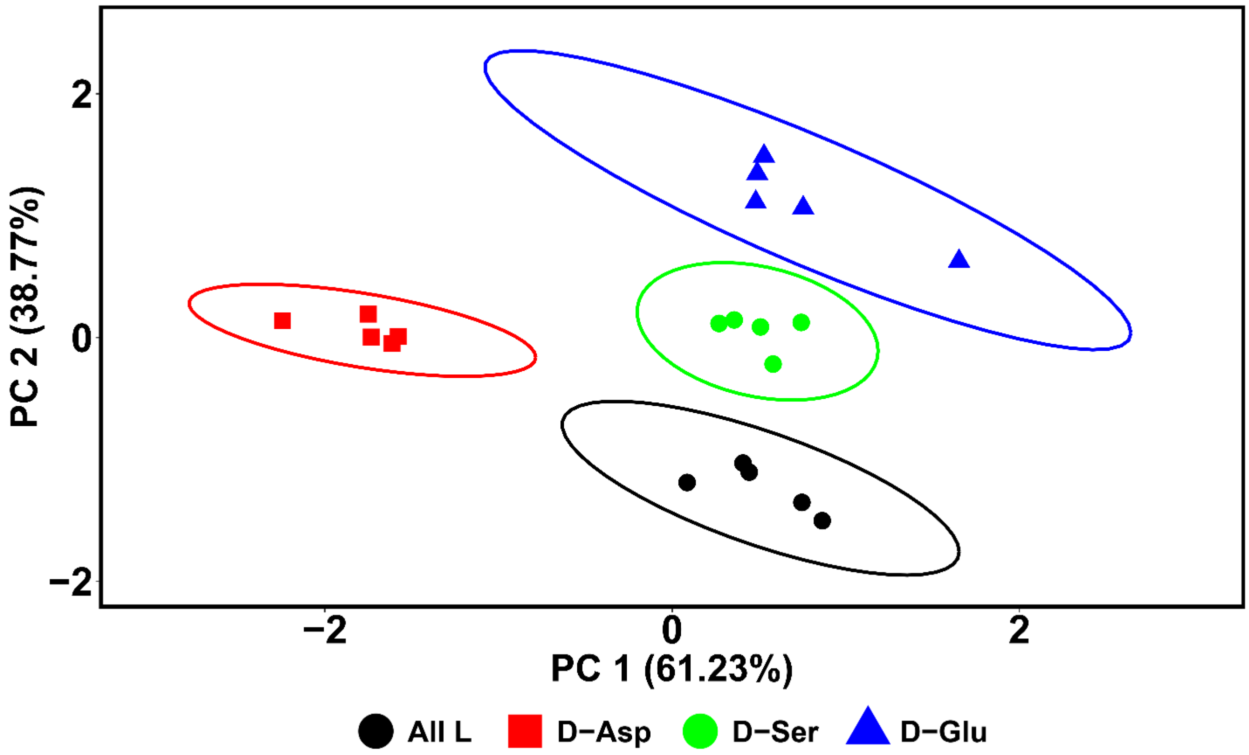

Figure *S*-28. The PCA score plot for the differentiation of 4 alphaB L/D isomers using the top two elements in SVM-RFECV rank list (Table *S*-6): **TCC•DTMI** and **TCC•2-DSMI**. Ellipses indicate 95% confidence.

6.3.4 SVM-RFECV of 16-Element pH 5 Array for 4 Asp Isomers

Table *S*-8. SVM-RFECV rank list of 16-element array in pH 5 buffer for classification of 4 alphaB Asp isomers: All L, D-Asp, L-isoAsp, and D-isoAsp forms.

Dye	Host	Rank	Select
**4-DSMI**	**TCC**	1	True
	**AMI**	5	False
	**AMD**	12	False
	**None**	14	False
**DTMI**	**TCC**	1	True
	**AMI**	7	False
	**AMD**	15	False
	**None**	6	False
**2-DSMI**	**TCC**	2	False
	**AMI**	8	False
	**AMD**	9	False
	**None**	4	False
**DQMI**	**TCC**	3	False
	**AMI**	13	False
	**AMD**	11	False
	**None**	10	False

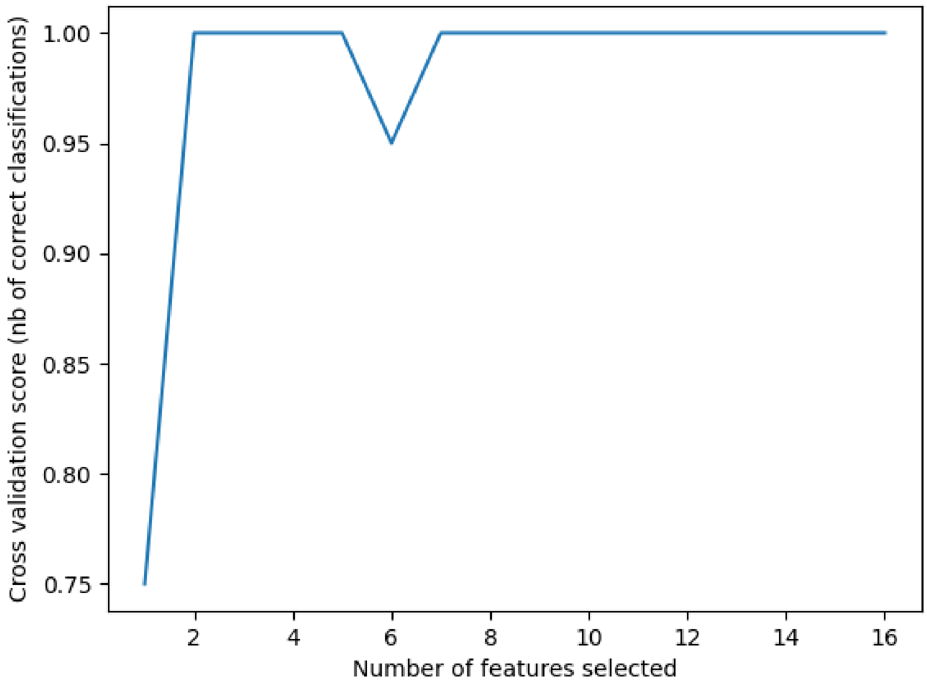

Figure *S*-29. The cross-validation scores correspond to the increasing numbers of features from the 16-element array for 4 alphaB Asp isomers classification.

Table *S*-9. The 3 repeated 4-fold cross-validation scores of SVM-RFECV selected 2 elements **TCC•DTMI** and **TCC•4-DSMI** from 16-element array in pH 5 buffer, with SVM as the estimator for classification of 4 alphaB Asp isomers: All L, D-Asp, L-isoAsp, and D-isoAsp forms.

**Evaluation Metrics**	**Score (standard deviation from 3 repeated running of the 4-fold cross validation)**
Accuracy	1.0000 (0.0000)
Sensitivity	1.0000 (0.0000)
Specificity	1.0000 (0.0000)
Precision	1.0000 (0.0000)
F1 Score	1.0000 (0.0000)
AUC	1.0000 (0.0000)

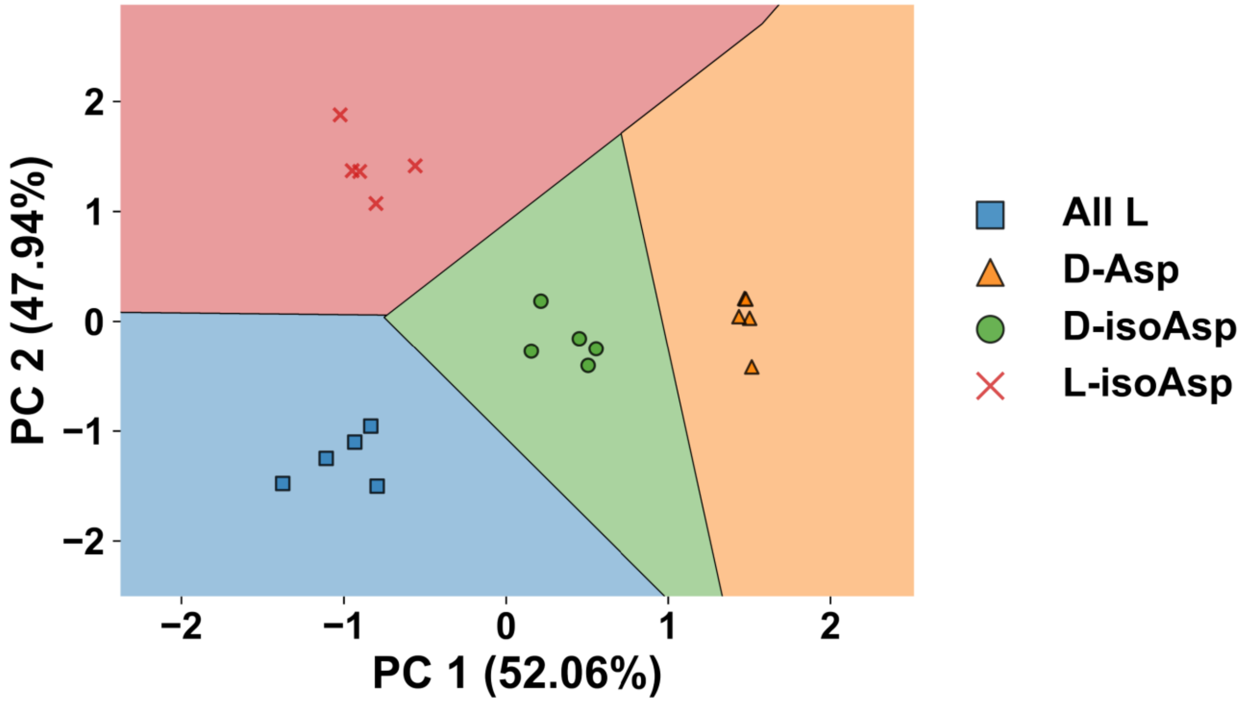

Figure *S*-30. The SVM decision region boundary plot for the classification of 4 alphaB Asp isomers using the PCA data of SVM-RFECV selected 2 elements **TCC•DTMI** and **TCC•4-DSMI** from 16-element array in pH 5 buffer.

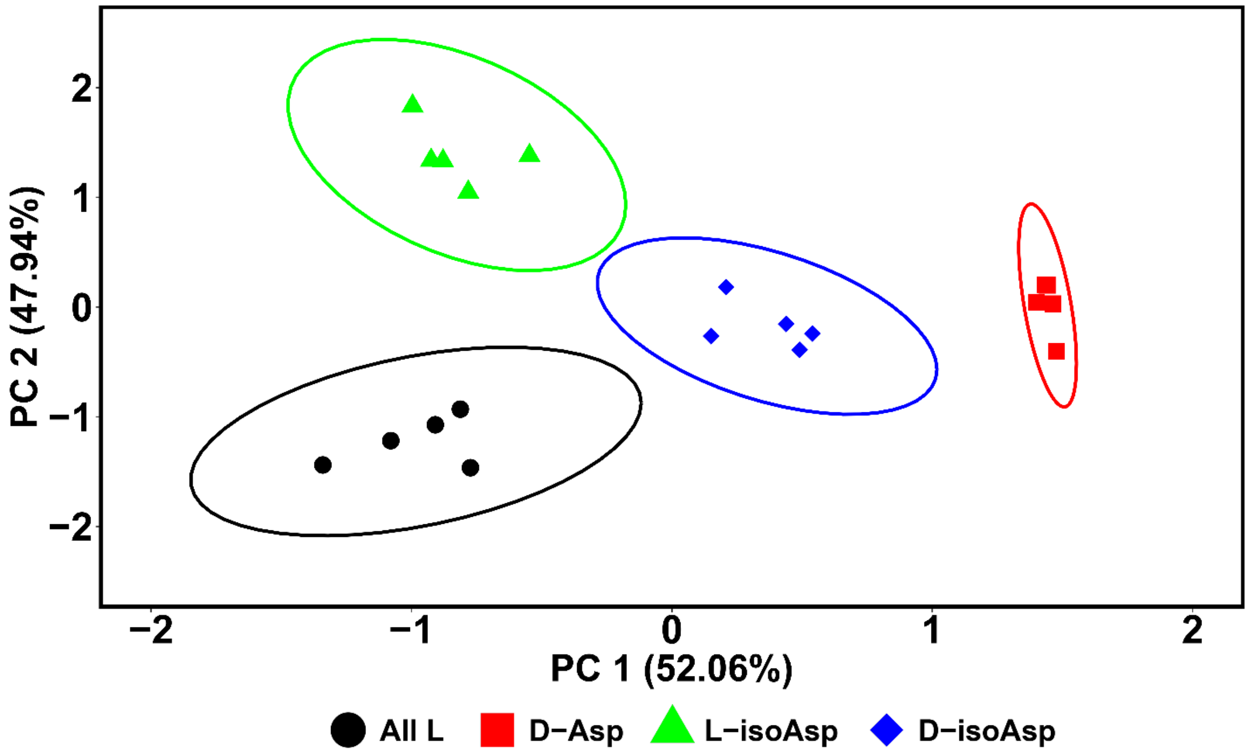

Figure *S*-31. The PCA score plot for the differentiation of 4 alphaB Asp isomers using the SVM-RFECV selected 2 elements **TCC•DTMI** and **TCC•4-DSMI** from 16-element array in pH 5 buffer. Ellipses indicate 95% confidence.

6.3.5 PCA Using Dye Only Elements From pH 5 Array

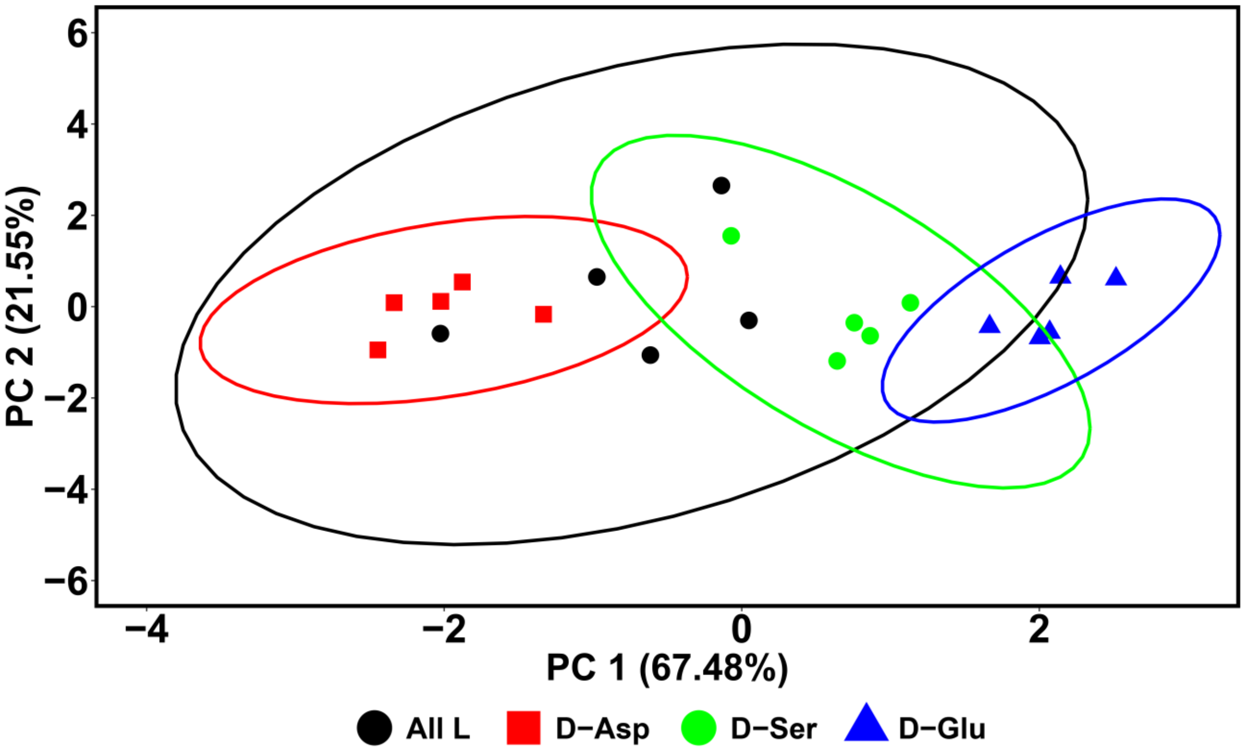

Figure *S*-32. The PCA score plot for the discrimination of 4 alphaB L/D isomers using the dye only elements: **4-DSMI**, **DTMI**, **2-DSMI**, and **DQMI**. Ellipses indicate 95% confidence.

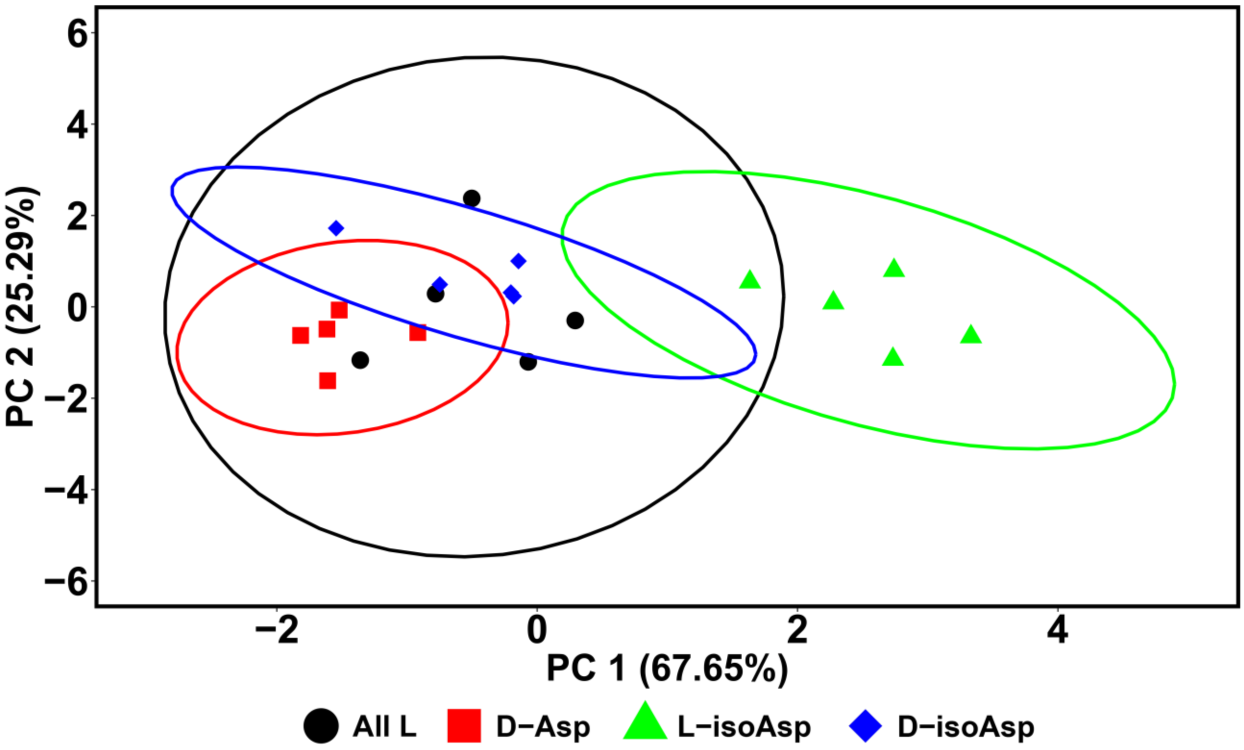

Figure *S*-33. The PCA score plot for the discrimination of 4 alphaB Asp isomers using the dye only elements: **4-DSMI**, **DTMI**, **2-DSMI**, and **DQMI**. Ellipses indicate 95% confidence.

6.4 Dye Titration of Trp (W)-Containing Abeta (Aβ) Mutant Peptides

Table *S*-10. The sequences of Trp (W)-containing Abeta mutant peptides. The mutation site was marked in red.

NO	Name	Sequence	a.a.	M.W.[a]	Net pH 7.4[a]	Net pH 5[a]	pI[a]	GRAVY[a]	Hydrophobicity[b]
1	Aβ-L	DAEFRHDSGY	10	1196.40	-2	-0.8	4.29	-1.61	13.14
2	Aβ-L S_mut_W	DAEFRHDWGY	10	1295.30	-2	-0.8	4.29	-1.62	23.82
3	Aβ-L G_mut_W	DAEFRHDSWY	10	1325.40	-2	-0.8	4.29	-1.66	24.20
4	Aβ-L Y_mut_W	DAEFRHDSGW	10	1219.10	-2	-0.8	4.29	-1.57	19.74

[a] Theoretical isoelectric point (pI), molecular weight (M.W., unit: g/mol), net charge under pH 7.4 or pH 5, and grand average of hydropathicity (GRAVY)[18] of peptides were calculated using Peptide Property Calculator on NovoPro website (https://www.novoprolabs.com/tools/calc_peptide_property).

[b] The hydrophobicity of peptides was obtained using Peptide Synthesis and Proteotypic Peptide Analyzing Tool on Thermo Fisher Scientific website (https://www.thermofisher.com/us/en/home/life-science/protein-biology/peptides-proteins/custom-peptide-synthesis-services/peptide-analyzing-tool.html), which is based on the 'Oleg V. Krokhin' Index and takes into account difficult combinations, such as avoiding too many of the same amino acid in the row and too many hydrophobic amino acids.

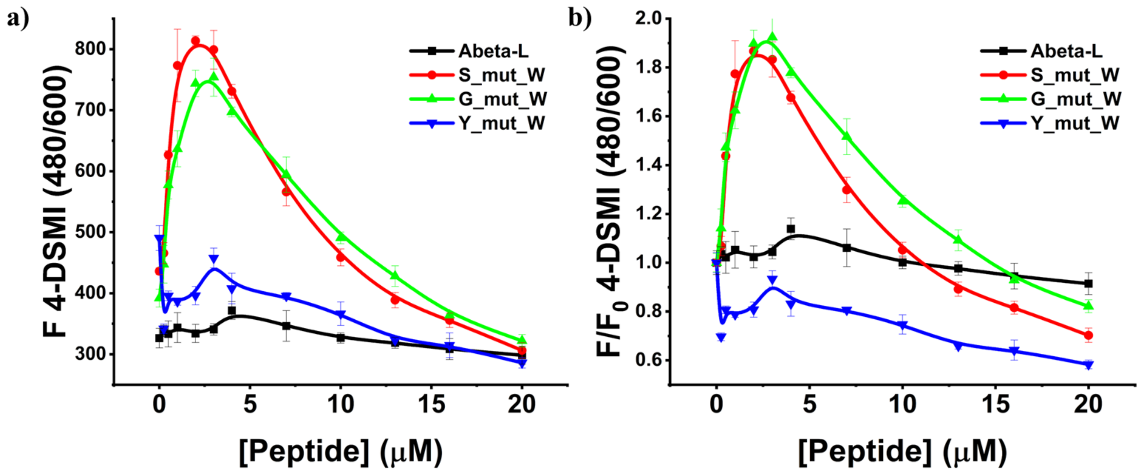

Figure *S*-34. a) Fluorescence and b) F/F_0 titration curves of 0.5 μM **4-DSMI** with increasing concentration 0 – 20 μM of Abeta-L, S_mut_W, G_mut_W, and Y_mut_W peptides in neutral H_2O, respectively.

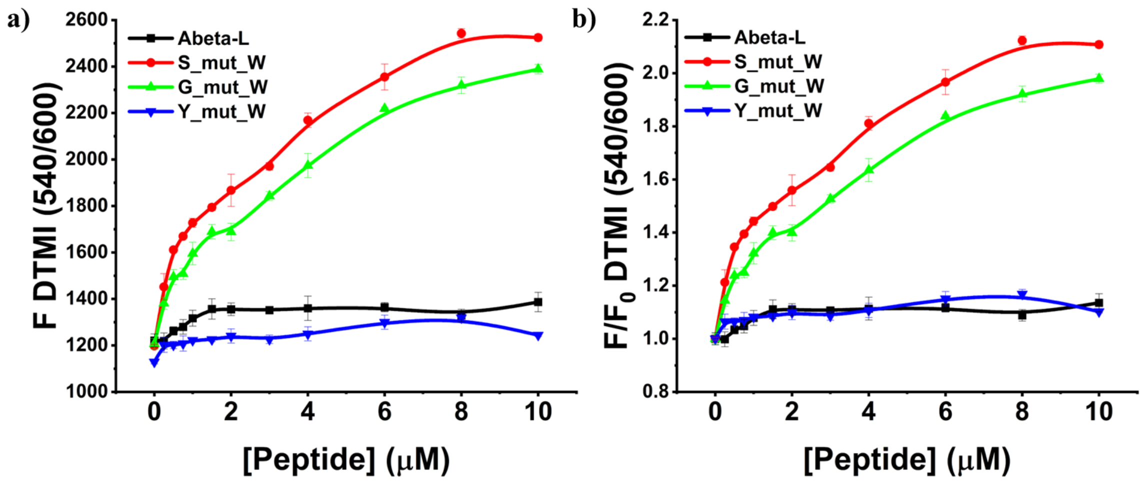

Figure *S*-35. a) Fluorescence and b) F/F_0 titration curves of 0.5 μM **DTMI** with increasing concentration 0 – 10 μM of Abeta-L, S_mut_W, G_mut_W, and Y_mut_W peptides in neutral H_2O, respectively.

7. References

1. S. M. Biros, E. C. Ullrich, F. Hof, L. Trembleau and J. Rebek, Jr., *J. Am. Chem. Soc.*, 2004, **126**, 2870-2876.
2. A. D. Gill, B. L. Hickey, S. Wang, M. Xue, W. Zhong and R. J. Hooley, *Chem. Commun.*, 2019, **55**, 13259-13262.
3. J. Chen, B. L. Hickey, Z. Gao, A. A. P. Raz, R. J. Hooley and W. Zhong, *ACS Sens.*, 2022, **7**, 2164-2169..
4. Y. Liu, M. Mettry, A. D. Gill, L. Perez, W. Zhong and R. J. Hooley, *Anal. Chem.*, 2017, **89**, 11113-11121.
5. J. Chen, A. D. Gill, B. L. Hickey, Z. Gao, X. Cui, R. J. Hooley and W. Zhong, *J. Am. Chem. Soc.*, 2021, **143**, 12791-12799.
6. S. U. Maier, A. B. Makwana and Todd A. Hare, *Neuron*, 2015, **87**, 621-631.
7. J. Wang, R. M. Wolf, J. W. Caldwell, P. A. Kollman and D. A. Case, *J. Comput. Chem.*, 2004, **25**, 1157-1174.
8. A. Jakalian, D. B. Jack and C. I. Bayly, *J. Comput. Chem.*, 2002, **23**, 1623-1641.
9. J. Chen, C. L. Brooks and J. Khandogin, *Curr. Opin. Struct. Biol.*, 2008, **18**, 140-148.
10. D. H. Herce, T. Darden and C. Sagui, *J. Chem. Phys.*, 2003, **119**, 7621-7632.
11. D. R. Roe and T. E. Cheatham, III, *J. Chem. Theory Comput.*, 2013, **9**, 3084-3095.
12. B. R. Miller III, T. D. McGee, Jr., J. M. Swails, N. Homeyer, H. Gohlke and A. E. Roitberg, *J. Chem. Theory Comput.*, 2012, **8**, 3314-3321.
13. Y. A. Lyon, G. M. Sabbah and R. R. Julian, *J. Proteome Res.*, 2017, **16**, 1797-1805.
14. E. E. Hubbard, L. R. Heil, G. E. Merrihew, J. P. Chhatwal, M. R. Farlow, C. A. McLean, B. Ghetti, K. L. Newell, M. P. Frosch, R. J. Bateman, E. B. Larson, C. D. Keene, R. J. Perrin, T. J. Montine, M. J. MacCoss and R. R. Julian, *J. Proteome Res.*, 2022, **21**, 118-131.
15. A. Watanabe, K. Takio and Y. Ihara, *J. Biol. Chem.*, 1999, **274**, 7368-7378.
16. T. R. Lambeth, D. L. Riggs, L. E. Talbert, J. Tang, E. Coburn, A. S. Kang, J. Noll, C. Augello, B. D. Ford and R. R. Julian, *ACS Cent. Sci.*, 2019, **5**, 1387-1395.
17. H.-T. Wu and R. R. Julian, *Analyst*, 2020, **145**, 5232-5241.
18. J. Kyte and R. F. Doolittle, *J. Mol. Biol.*, 1982, **157**, 105-132.
19. Y. Tao and R. R. Julian, *Anal. Chem.*, 2014, **86**, 9733-9741.
20. N. Fujii, H. Sakaue, H. Sasaki and N. Fujii, *J. Biol. Chem.*, 2012, **287**, 39992-40002.
